# Osmotic modulation of chromatin impacts on efficiency and kinetics of cell fate modulation

**DOI:** 10.1038/s41598-018-25517-2

**Published:** 2018-05-08

**Authors:** A. F. Lima, G. May, J. Díaz-Colunga, S. Pedreiro, A. Paiva, L. Ferreira, T. Enver, F. J. Iborra, R. Pires das Neves

**Affiliations:** 1 UC-Biotech, CNC - Center for Neuroscience and Cell Biology, University of Coimbra, 3060-197 Cantanhede, Portugal; 2 Faculty of Science and Technology, University Nova of Lisbon (MIT-Portugal PhD Program), 2829-516 Caparica, Portugal; 3 University College London, Gower Street, London, WC1E 6BT UK; 4 Centro Nacional de Biotecnología, CSIC. Darwin 3, Campus de Cantoblanco, 28049 Madrid, Spain; 5 Unidade de Gestão Operacional de Citometria, Centro Hospitalar e Universitário de Coimbra, 3000-075 Coimbra, Portugal; 6 Faculty of Medicine, University of Coimbra, 3004-504 Coimbra, Portugal; 7 Institute for Interdisciplinary Research, University of Coimbra, 3030-789 Coimbra, Portugal; 8 Coimbra Institute for Clinical and Biomedical Research, Faculty of Medicine,University of Coimbra, 3004-504 Coimbra, Portugal

## Abstract

Chromatin structure is a major regulator of transcription and gene expression. Herein we explore the use of osmotic modulation to modify the chromatin structure and reprogram gene expression. In this study we use the extracellular osmotic pressure as a chromatin structure and transcriptional modulator. Hyposmotic modulation promotes chromatin loosening and induces changes in RNA polymerase II (Pol II) activity. The chromatin decondensation opens space for higher amounts of DNA engaged RNA Pol II. Hyposmotic modulation constitutes an alternative route to manipulate cell fate decisions. This technology was tested in model protocols of induced pluripotency and transdifferentiation in cells growing in suspension and adherent to substrates, CD34^+^ umbilical-cord-blood (UCB), fibroblasts and B-cells. The efficiency and kinetics of these cell fate modulation processes were improved by transient hyposmotic modulation of the cell environment.

## Introduction

Osmotic stress can alter the role of molecular players involved in transcription itself and impact in the epigenetic state of the cell. As a consequence, the RNA repertoire changes during and after stress stimuli. Permanent hyposmotic stress has been shown to promote the upregulation of specific lncRNAs that exert functions in rRNA gene silencing^1^. Additionally, this type of modulation can change chromatin topology by biophysical distortion of the nucleus and it may alter gene expression; although direct experimental evidence for this is still lacking^2–4^. The osmotic pressure can be a biophysical stressor that promotes water entrance and induces cell and nuclear size changes, with alterations in chromatin structure^2,5–8^. These features are crucial for cell state maintenance and fate decisions. Interestingly, the first nuclear reprogramming experiments by Gurdon and colleagues (1968), have shown a rapid nuclear swelling, a dispersion of chromosomes and chromatin, the entry of protein and the induction of DNA and RNA synthesis^9^ in a sequential temporal order, after the nuclear injection into the egg cytoplasm. This type of experiments also suggests that the cytoplasm harbours one or more soluble factors that are diffusible, specify cellular identity, and can trigger transdifferentiation to other cell types. Could the osmotic environment of the egg help this process? Although in these seminal experiments the osmolarity of the nucleus and the egg at the time of injection is unknown, it is well described in the assisted reproduction field that the osmotic environment control is fundamental for successful fertilization. In intracytoplasmic sperm injection (ICSI) it is routine, to select the cells that perform best in a hyposmotic swelling test (HOST) which have been shown to lead to the formation of embryos with higher developmental potential^10^. On the other hand, it can be argued that even cells that have a low HOST score have the same fertility potential when the cell is delivered inside the cytoplasm and therefore HOST should be moot for ICSI cycles^10^. Could it be the case that HOST preconditions the sperm for chromatin decondensation facilitating the process later on? Indeed, chromatin transformations are widely accepted as major rate-limiting steps during cellular fate reprogramming^11–13^. There are several master transcription factors (TFs) capable of defining the cell state and these TFs have been used to trigger transdifferentiation across all major lineages (reviewed in^14^). At the apex of all cell types generated by TF overexpression, the induced pluripotent stem cells (iPSCs) have gained particular attention because they have the unique potential to generate all the adult cell types. The search for factors that boost the kinetics of reprogramming has found small molecules that impact on nucleosome structure, which constitutes an important barrier for RNA Pol II processivity and to the emergence of new transcription sites^11,15–17^.

In this study, we show that a transient hyposmotic shift promotes chromatin loosening and the recruitment of RNA Pol II to bind the cellular DNA. This novel methodology coupled to exogenous transcription factor expression may be used in all kinds of cellular fate reprogramming scenarios.

## Results

### Tailoring osmotic stimuli into a cell physiology modulation tool

**Figure 1:**
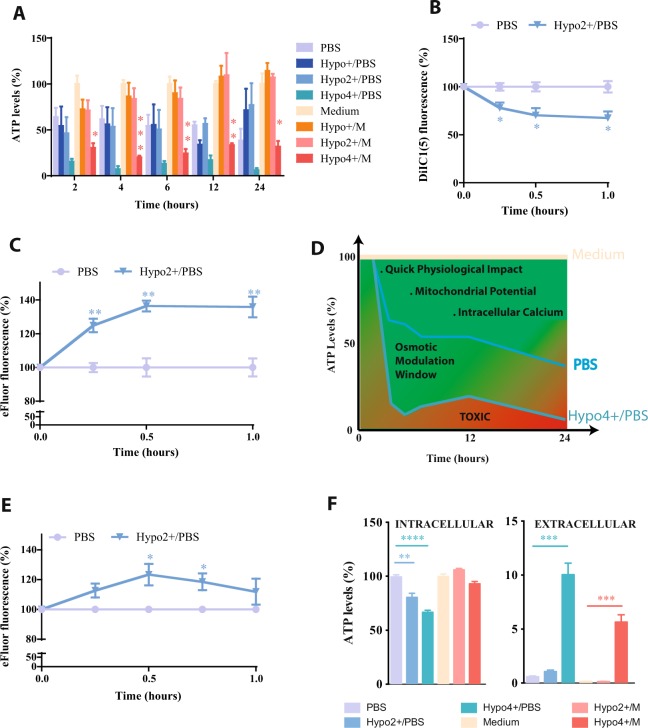
Impact of different osmotic environments in K562 cell physiology. (**A**) to (**C**) Effect of constant osmotic modulation on ATP levels (% normalised by medium condition), mitochondrial membrane potential (DiIC1(5)) and intracellular free calcium levels (e-Fluor) (% of fluorescence signal normalised to the control). (**D**) Schematic representation of the safety of the PBS-based hyposmotic modulation strategy. This scheme is derived from (**A**) and highlights the deleterious effect that even PBS by itself has in permanent culturing conditions. (**E**) and (**F**) Effect of transient osmotic modulation (15 minutes) on intracellular free calcium levels (e-Fluor) and intracellular vs extracellular ATP levels (% normalised to the medium/PBS condition). The different osmotic modulation protocols are described in the legend of each graph. The variations presented are the mean value ± SEM (n ≥ 3) and the changes are statistically significant at the time points highlighted in the graphs (*p value < 0.05; **p value < 0.01; ***p value < 0.001; ****p value < 0.0001).

First, a systematic evaluation of the impact of hyposmotic pressure in cell physiology was done. For that, PBS- (hypo/PBS) or media-based (hypo/M) cocktails (Supplementary Table S1) were used in prolonged (up to 24 hours) or transient (15 minutes) protocols and with variable degrees of dilution of the PBS or media (as detailed in Supplementary Table S1). A safety threshold was observed in K562 cell line (used as a proxy for cord-blood mononuclear cells) for hyposmotic modulation based on the analyses of the following parameters: forward side scatter (FSC), as an indirect measure of cell size^18^ (Supplementary Fig. S1A); adenosine triphosphate (ATP) levels, as a measure of cell viability (Fig. 1A); and production of reactive oxygen species (ROS) (Supplementary Fig. S1B), mitochondrial membrane potential (Fig. 1B) and intracellular free calcium (Fig. 1C), as general physiologic readouts. Short term mild hyposmotic modulation (hypo+/M, hypo2+/M, hypo+/PBS and hypo2+/PBS) is safe in K562 cells (Fig. 1D). During the first hour of exposure to mild PBS-hypotonic conditions there is a decrease in ATP (Fig. 1A), mitochondrial membrane potential (Fig. 1B) and an increase in intracellular calcium (Fig. 1C) which does not translate into significant oxidative stress (Supplementary Fig. S1B and S1C) and proves the point that cells react quickly in these conditions. The same assay, done with medium-based osmotic conditions, shows no significant alterations (Supplementary Fig. S1D to S1E). The time of exposure to the osmotic modulation is a determinant factor. When the cells are exposed to mild PBS-based conditions during 15 minutes and then return to an isosmolar media, they quickly reach normal calcium levels (Fig. 1E). This behaviour is conditioned by the presence or absence of calcium in the extracellular environment and the need to mobilize calcium from the intracellular stores (e.g. endoplasmic reticulum and mitochondria). This effect is observed when the previous experiment is done in a calcium-containing culture medium where the response is sustained for a longer period of time (Supplementary Fig. S1F). Under PBS-based conditions, because calcium is absent in the extracellular environment, there is an increased physiological impact (Fig. 1E). Another important effect of the hyposmolar modulation is the quick drop in ATP levels (Fig. 1F). This effect is not only due to a metabolic effect at the level of synthesis but is also due to extrusion of ATP from the cell (Fig. 1F). This is a clear effect for low osmolarity where more than 15% of the ATP of the cell is extruded after 15 minutes of modulation (Fig. 1F). This observation is in agreement with the ATP extrusion hypothesis, where the release of ATP from cells after hyposmotic modulation occurs^19^. Moreover, in parallel to the increased extracellular ATP level, after exposure to hypo4+/PBS (Fig. 1F) there is a significant decrease of the intracellular ATP (Fig. 1F). The decrease in intracellular levels of ATP and increase in intracellular calcium is already described in the literature for several cell types^2,7,19,20^. UCB stem cells are particularly sensitive to the osmotic environment during collection and cryopreservation^21,22^, and these changes can influence their performance and fate. CPDA-1, a calcium-chelator routinely used for UCB collection, can induce a hyperosmotic and acidic environment when present in high concentration in collected blood. Our studies have shown that the change in osmolarity during UCB collection can have an impact in survival and stem cell potential (Supplementary Fig. S2).

This safety threshold described for K562 cells has to be determined in different cells subjected to hyposmotic modulation. A similar assessment of cell behaviour was done for human fibroblasts and the results differ from the ones obtained in K562 cells (Supplementary Fig. S2). Fibroblasts are more sensitive to the hyposmotic modulation and the adhesion to the cell culture substrate is affected. In long-term PBS-based hyposmotic stimulus as well as after one hour in hypo2+/PBS, the cells display a round morphology instead of their characteristic elongated morphology (Supplementary Fig. S3H). In long-term PBS-based hyposmotic conditions, fibroblasts display a morphology that resembles the anoikis process^23^ (Supplementary Fig. S3H), where the prolonged absence of calcium has a crucial role.

Furthermore, the osmotic effect on intracellular calcium regulation enlists mitochondria as one of the targeted organelles^24^. Using time-lapse microscopy and cells carrying fluorescent mitochondria (MitoGreen HeLa cells carrying EGFP-mitochondrial localization signal inside the mitochondrial matrix, adapted from^25^) we observed an immediate impact of hyposmotic modulation on mitochondrial morphology (Supplementary Fig. S8D). Modulation with hypo2+/PBS induces the immediate appearance of a spotted mitochondrial phenotype which is almost completely reverted after some minutes, when mitochondria tend to regain the network features (Supplementary Fig. S8D).

### Hyposmotic modulation changes the structure of the chromatin and the transcriptional activity of RNA Pol II

**Figure 2:**
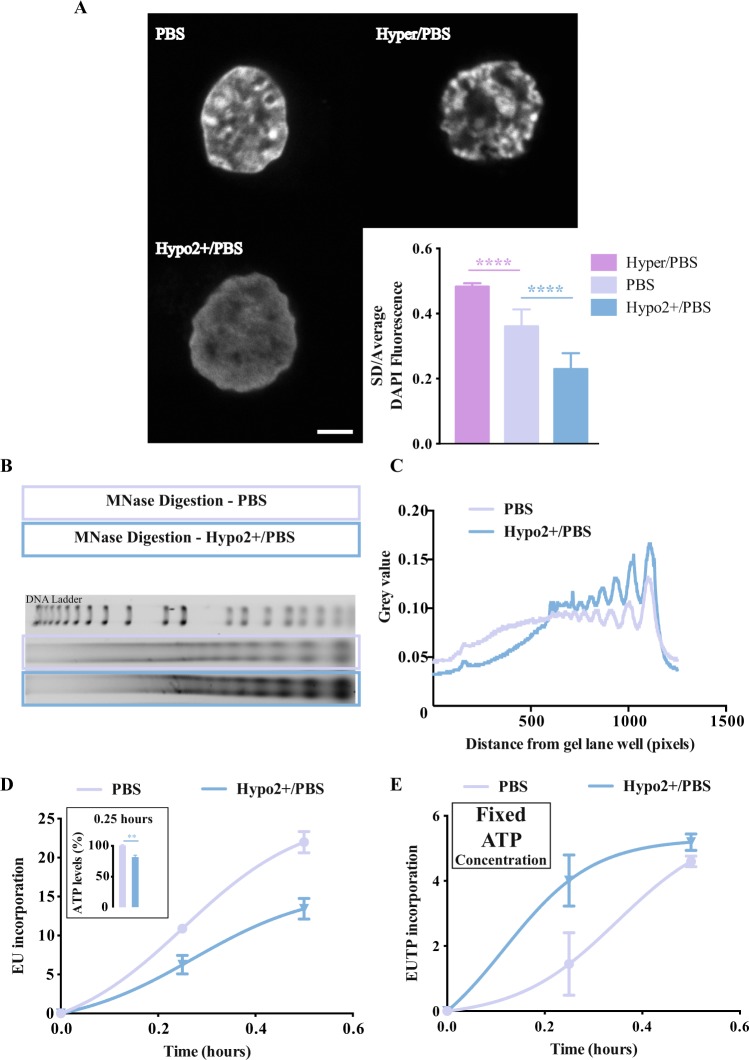
Impact of different osmotic environments in nuclear structure, chromatin organization and transcription. (**A**) Confocal imaging with DAPI stained K562, fixed and imaged straight after exposure to different osmotic modulations (scale bar = 2 μm) and respective coefficient of variance of the DAPI staining (approximately 100 cells per condition were analysed). (**B**) DNA fragmentation pattern after MNase digestion (150 gel units) using K562 cells that were transiently in different osmotic conditions (cropped lanes from the original gel image provided in Supplementary Fig. S6C). (**C**) Representative histogram of the DNA fragmentation pattern of MNase digestion presented in B. The digestion pattern was analysed in Image J. (**D**) Effect of constant hyposmotic modulation on transcriptional activity assessed by EU incorporation in K562 cells. A nonlinear regression was done to each condition. Insert showing the drop in ATP levels for the same time point. (**E**) Effect of transient hyposmotic modulation on transcriptional activity assessed by EUTP incorporation in K562 cells. This EUTP protocol is run under fixed/non-limiting ATP concentration conditions. A nonlinear regression was done to each condition. The different osmotic modulation protocols are described in the legend of each graph. The variations presented are the mean value ± SEM (n ≥ 3) and the changes are statistically significant at the time points highlighted in the graphs (****p value < 0.0001).

Here we explore the impact of transient hyposmotic swelling on nuclear architecture and chromatin structure. Previous studies have shown that altering the physical environment of the chromatin impacts the overall chromatin structure and transcriptional activity^26–28^. Chromatin structure can be assessed in the microscope. Heterochromatic regions correspond to brighter fluorescence spots of DAPI staining and are inactive areas of transcription^4^. On the other hand, euchromatin corresponds to less intense and more homogeneous regions of DAPI staining. These are considered transcriptionally active. Figure 2A shows that the exposure of cells to a hypertonic solution increases the heterochromatin spot density and intensity. On the other hand, the exposure to hyposmotic solutions induces a more homogeneous nuclear DAPI staining, with less heterochromatic spots (Fig. 2A). This can be quantified using as a proxy the coefficient of variation (CV = Standard Deviation/average intensity) of the DAPI fluorescence signal. A lower CV of DAPI staining reflects a more homogeneous chromatin (decondensed chromatin) and high CV denotes the presence of condensed chromatin. Cells exposed to the hyposmotic condition show a low CV and the opposite effect was seen in hyperosmotic condition (Fig. 2A graph). These alterations are proportional to the intensity of the stimulus (hypo/PBS, hypo+/PBS, hypo2+/PBS, hypo4+/PBS) (Supplementary Fig. S4A). The same experiments done in UCB derived mononuclear cells show similar trends in DAPI staining (Supplementary Fig. S4B). In addition, similar experiments in HeLa cells have shown an oscillatory behaviour of the nuclear area and the nuclear DAPI staining intensity (Supplementary Fig. S4C to S4F).

To study the structure of the chromatin in more detail nuclease enzymatic digestion was used. More accessible chromatin is easier to digest^29^ and this is seen after transient hyposmotic modulation. The pattern of digestion with micrococcal nuclease (MNase) denotes differences in chromatin accessibility after hyposmotic modulation that can be observed in Fig. 2B and quantified in Fig. 2C. Additionally, the digestion pattern with deoxyribonuclease I (DNase I) follows the same trend of increased chromatin accessibility after transient hyposmotic modulation (Supplementary Fig. S6D). Open chromatin is permissive to transcription^29,30^ and if the hyposmotic modulation induces an increased euchromatic content this should be correlated with increased production of RNA. This increase cannot be observed in a classic transcriptional assay, using the artificial precursor EU, where the hyposmolarity seems to induce a reduction in EU incorporation into the newly synthetized RNA (Fig. 2D). This decrease in production of RNA is associated with the reduction in [ATP] in the hyposmotic environment (Fig. 1A,F). We have previously shown that transcription speed is influenced by fluctuations on intracellular ATP levels^31^. When a similar analysis was done this time with EUTP and with constant/ non-limiting [ATP] (similar to^31^) it was possible to observe an increase in transcription under hyposmotic conditions and the opposite in hypertonicity. The incorporation of EUTP into the newly synthesised RNA was 54% higher after a transient hyposmotic modulation, during the first 50 min of assay (Fig. 2E). Furthermore, these results are consistent with the increase of RNA Pol II phosphorylated at Ser2 (a putative marker for RNA Pol II engaged in transcription), detected by western blot immediately after the hyposmotic modulation (Supplementary Fig. S6E). Analyses of the acetylation of histone H4 at lysine16 (reporter of transcription activity) in UCB cells have shown that hyposmotic modulation induces variable acetylation patterns depending on the intensity of the stimulus (Supplementary Fig. S6F). The high variability of these measurements suggests that this labelling is fluctuating quickly. Therefore, the H4K16ac profile was evaluated in HeLa cells (for practical reasons) along the time. After hyposmotic modulation the changes in the acetylation profile of H4K16 seem to accompany the trend of increased transcriptional profile overtime, although an initial drop is noticed right after the hyposmotic modulation (possible ATP effect already discussed) (Supplementary Fig. S6G). Interestingly, right after the hyposmotic modulation it was not possible to observe changes in other relevant histone modifications suggesting that there are different kinetic regimes for different histone modifications (Supplementary Fig. S6E).

The impact of osmolarity on chromatin structure and RNA Pol II activity is not specific for cells in suspension. Indeed, cells growing attached to substrates, show altered chromatin structure and kinetics of RNA production after hyposmotic modulation with different responsiveness when compared to suspension cells. Using an adherent cell line and a classic BrUTP transcription assay it was possible to observe similar transcriptional changes described above for K562 cells (Supplementary Figs S6A and S6B).

**Figure 3:**
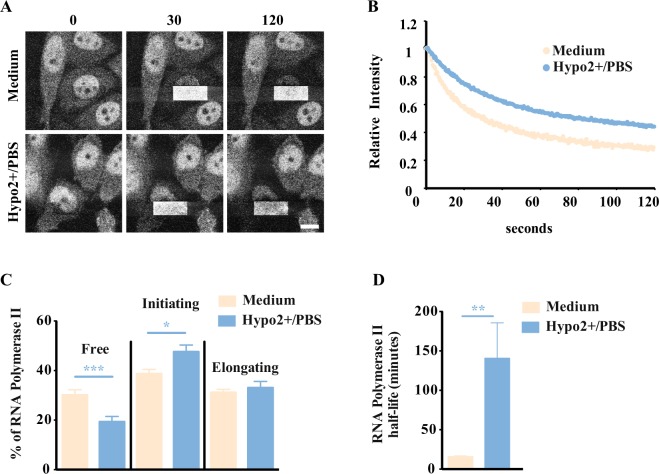
Impact of different osmotic modulations in RNA polymerase II dynamics. (**A**) Fluorescence loss in photobleaching (FLIP) was performed in the LSM 710 (Carl Zeiss) confocal microscope with the stage heated at 39 °C with the CHO RNA Pol II-EGFP cell line. A rectangle of half of each nucleus was selected where 100% laser power was applied, in order to bleach all the fluorescent molecules in these rectangles. This operation was repeated approximately every 5 seconds for a period of 900 seconds originating the decay of fluorescence in the unbleached half. (**B**) Representative decay curves of 2 single cells. The EGFP intensity values in the unbleached area were evaluated with ImageJ and fitted to three populations with an exponential decay $({\boldsymbol{f}}={\boldsymbol{a}}\times {\bf{\exp }}(\,-\,{\boldsymbol{b}}\times {\boldsymbol{x}})+{\boldsymbol{c}}\times {\bf{\exp }}({\boldsymbol{d}}\times {\boldsymbol{x}})+{\boldsymbol{g}}\ast {\bf{\exp }}(\,-\,{\boldsymbol{h}}\times {\boldsymbol{x}})$; R^2^ > 0.99:: one free form, one bound to DNA but not fully engaged, and another fully engaged in transcription (see model in Fig. S5 for further details) (**C**) Percentage of different RNA polymerase II forms of the transcription cycle. Data derived from the FLIP analysis allows to estimate the dissociation constants and the percentage of the different populations from decay curves like the ones in B. (**D**) RNA polymerase II half-life data derived from the FLIP analyses showing the impact of hyposmolarity on the speed of transcription. Higher half-life of RNA pol II is indicative of a slower transcription activity. The different osmotic modulation protocols are described in the legend of each graph. The variations presented are the mean value ± SEM (n ≥ 3) and the changes are statistically significant at the time points highlighted in the graphs (*p value < 0.05; **p value < 0.01; ***p value < 0.001).

To complement the analyses of the impact that hyposmotic modulation has on transcription activity in attached cells the dynamics of fluorescent RNA pol II (method used in^31,32^) were measured. In hypo2+/PBS conditions (Fig. 3A,B) there is a significant decrease in the free form of RNA Pol II and a significant increase in the initiating form of the polymerase (Fig. 3C). Although the hyposmolarity (hypo2+/PBS) does not seem to have an impact in the percentage of RNA Pol II in transcriptional elongation (Fig. 3C) it does promote a significant increase in the half-life of RNA Pol II engaged in transcription elongation (Fig. 3D). In this experiment, the average RNA Pol II half-life in the hyposmotic condition (hypo2+/PBS) is approximately 140 minutes which is significantly increased when compared to the isosmolar condition ∼16 minutes (Fig. 3D). These kinetics are in agreement with previous studies that assume that a transcription unit in this model cell line has the same length as a human gene (median length ∼14 kbp;^33^), and a polymerization rate of 1.1–2.5 × 10^3^ nucleotides/min^34^, meaning that a typical transcription unit would be copied in 6–13 min in isosmolar conditions^35^.

### Hyposmotic modulation induces a new transcriptional network with increased binding of RNA pol II

Changes in the transcription kinetics suggest that the loading of RNA Pol II on the DNA after hyposmotic modulation may be different. This possibility was studied by RNA Pol II chromatin immunoprecipitation and sequencing assays (ChIP-Seq). The binding pattern of the different forms of RNA Pol II: total, initiating (PhosphoS5) and elongating (PhosphoS2) was evaluated by using specific antibodies for the phosphorylated forms of the RNA Pol II CTD, after transient exposure to hyposmolarity (hypo2+/PBS). These analyses were done in two different time points, one immediately after the transient hyposmotic modulation (hypo2+/PBS; 15 min.) and another one, after 1 hour of recovery in complete growth medium. In the hyposmotic condition, a higher amount of DNA was recovered after the ChIP procedure, suggesting an increased binding of RNA Pol II to cellular chromatin (Supplementary Fig. S8).

**Figure 4:**
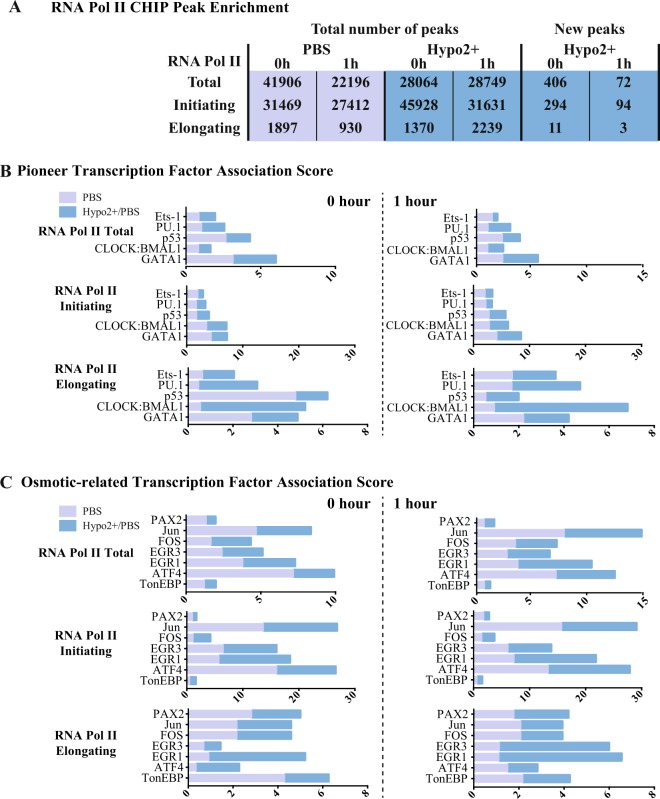
Impact of hyposmotic modulation in RNA Pol II binding profile and transcription. (**A**) Total number of ChIP-Sequencing peaks detected after background noise subtraction, done in K562 cells. (**B**) and (**C**) Relevant transcription factors association scores. Association scores were computed with PASTAA (http://trap.molgen.mpg.de/cgi-bin/pastaa.cgi) as an *in silico* approach to infer the binding/affinity of transcription factors involved in the regulation of genes enriched in the ChIP-Seq assays for the different forms of RNA Pol II.

The ChIP-Seq data shows that the hyposmotic modulation has an impact on the RNA Pol II binding profile to chromatin (Fig. 4A). The detection of peaks in each condition shows that the number of RNA Pol II PhosphoS5 peaks is increased almost 1.5-fold right after the modulation when compared to the PBS control (Fig. 4A). Moreover, after 1 h of recovery from the transient hyposmotic stimulus, the chromatin is enriched for the RNA Pol II PhosphoS2 binding (Fig. 4A). In order to validate the Chip-seq data set, bioinformatics analyses were undertaken based on the principle that there should be a high degree of agreement between the peak IDs obtained after Chip with different antibodies for RNA pol II for the same condition. Indeed, in all samples (control and hypo), at both 0 h and 1 h, roughly 85-95% of the features that appeared in the RNA Pol II PhosphoS5 lists were also present in the Total RNA pol II ones (Supplementary Fig. S7). To further complement these results, a very stringent analysis was performed to determine whether the hyposmotic modulation was promoting binding of RNA Pol II forms to new binding sites or increasing the binding and/or recovery of RNA Pol II at the same binding sites. For this analysis, we compared the peaks detected, in the samples PBS versus hypo2+/PBS both normalised to the respective IgG controls. Afterwards, we filtered them to have a fold enrichment higher than 30 or 15 (in the case of RNA Pol II PhosphoS2) and a p-value lower than 1 × 10^−9^. This analysis generated a list of regions called “new peaks” that were only present in the hypo2+/PBS condition. Therefore the hyposmotic modulation promotes the binding of RNA Pol II to new sites of the cellular genome (Fig. 4A).

This data demonstrates that after a transient hyposmotic shock, cells have a distinct RNA Pol II binding profile that can enable the cell to change the transcriptional network. Within the list of genes in the neighbourhood of the newly bound sites by RNA Pol II, there are numerous targets associated with chromatin modifications, transcription control, calcium homoeostasis, potassium channels, kinases, several RNA genes (non-coding RNA, long-non-coding RNA, antisense RNA and others), and microRNAs (Supplementary Table S2). Besides all the chromatin and transcription associated hits there is the interesting effect in potassium channels and calcium homeostasis which should be expected in response to a hyposmolar stimulus that induce the entrance of water and ionic balance compensation fluxes.

With respect to water, there are a few examples in the literature of TF binding to DNA to be dependent on the specific hydration of DNA sequences. One example is the pioneer factor PU.1. Therefore, we decided to evaluate *in silico* using a bioinformatics approach based on the Chip-Seq results, if this is a general characteristic of pioneer TFs. TFs that are affected by the levels of hydration of DNA should have their binding affinities influenced by the hyposmotic modulation and one readout would be the binding of RNA Pol II to specific genes in their regulatory network. Additionally, as there is no extensive list of pioneer factors described in the literature, we decided to evaluate the impact of hyposmolarity on the ability of pioneer TFs to bind the DNA using the list of pioneer TFs available in^36^. We used the ChIP-Seq results to evaluate the different capacity of recognition and binding of RNA Pol II under hyposmotic modulation. To identify the TFs that might promote that specific RNA Pol II binding profile we used PASTAA, a tool from Max Planck Institute (http://trap.molgen.mpg.de/cgi-bin/pastaa.cgi). This method detects TFs that are associated with particular functional categories of genes. PASTAA stands for: predicting associated transcription factors from annotated affinities, because as a first step it ranks all genes by the predicted affinity of the TF to the genes’ promoters. We used the association scores computed by PASTAA (-log of the most significant hypergeometric p-value) as a readout for the binding/affinity probability of a TF to be involved in the regulation of a set of genes enriched in the ChIP-Seq of the different forms of RNA Pol II. When the list of enriched genes in the ChIP-Seq experiment of RNA Pol II Total, PhosphoS5 and PhosphoS2 forms were analysed in PASTAA we could only get scores for the pioneer TFs: PU.1, GATA1, CLOCK:BMAL1 and p53, as presented in Fig. 4B, probably meaning that the other pioneer TFs are not expressed in K562 cells. For instance, the GATA4 promoter is hypermethylated in K562 cells and the levels of expression of GATA4 are low^37^. If this is the case it is expectable that genes that are regulated by this pioneer TF are not enriched for RNA Pol II binding as our analyses show.

It is interesting to note that the great majority of the TFs show higher scores in the hyposmotic condition than in the PBS condition (Fig. 4B). Also, most TFs show a drop at 1 hour, except for PU.1, Ets1 and CLOCK:BMAL1. From these, only PU.1 has been shown to have specific needs of hydration in its binding sequences^38,39^. Indeed, in the hyposmotic modulatory condition PU.1 shows a higher association score when compared with Ets1 (non-pioneer TF of the same family of PU.1) for total RNA Pol II particularly at time 1 hour (Fig. 4B). This is mainly due to the contribution of the elongating form of RNA Pol II and suggests that PU.1 may be a co-factor for transcription in the body of genes that are more expressed in the hyposmotic condition. For CLOCK:BMAL1 there is no description on hydration influencing binding affinities for this pioneer TF, but it has been recently shown that cellular mechano-environment regulates these circadian clock genes in the mammary and pulmonary systems^40^. Under a softer mechano-environment these genes are up-regulated. What our results suggest is that CLOCK:BMAL1 has a high association score with the hyposmotic condition particularly again for the elongation form of RNA Pol II (Fig. 4B).

Next we tried to validate our *in silico* analyses looking at genes that are referenced in the literature as being affected by hyposmotic modulation. This was a difficult task as most of the literature is available for hypertonic stress. Therefore, we looked at the Human Osmotic Stress RT2 Profiler PCR Array from SABiosciences for a reference on validated TFs that are affected by osmotic stress. This array profiles the expression of 84 key genes involved in the cellular response to changes in osmolarity and identifies the TFs TonEBP, ATF4, DDIT3, EGR1, EGR3, FOS, JUN, PAX2, SNAI1, TP53 and ZFP36L1 as important regulators of the osmotic stress response. Based on this list we ran our ChIP-Seq data in PASTAA and looked for association scores of these TFs in hyposmotic or PBS conditions and the respective results are presented in Fig. 4C. Again, most TFs show higher scores in the hyposmotic condition and these are maintained 1 hour after stimuli. No association scores were found for SNAI1, DDIT3 and ZFP66L1 probably meaning that these proteins are not expressed in K562 cells. The most interesting effects of the hyposmotic condition were seen in the gene regulatory networks of EGR1, EGR3, Jun and ATF4. The presence of EGR1 was an interesting hit because it is reported in the literature as having activity impairment with increasing amounts of osmolytes^41^. One of the first events after the hyposmotic stimulus is the exit of osmolytes from the cell to balance the extracellular hyposmotic environment. Also, under hyposmotic stimulus, osmolytes leave the interaction pockets of proteins; so this type of effect renders the EGR1 freedom to interact with the DNA. To further explore this *in silico* approach and understand if these TFs could be associated with the “new peaks” that are significantly enriched in the hyposmotic modulation condition we ran the list of “new peaks” for RNA Pol II PhosphoS5 (0 and 1 hours) in PASTAA. Surprisingly, immediately after the hyposmotic stimulus (0 h) eight out of the ten higher ranked TFs whose binding motifs were found to be associated with the “new peaks” ChIP-Seq data of the initiating form of RNA Pol II, belong to the superclass of “zinc-coordinating DNA-binding domains” (Supplementary Fig. S8C). This high prevalence of zinc-finger (ZF) TFs is not seen anymore 1 hour after hyposmotic modulation (Supplementary Fig. S8C) or in the Iso condition ChIP-Seq for RNA Pol II PhosphoS5 (where the percentage of ZF TFs in the top 10 is lower than 10% - data not shown). These results suggest that under hyposmotic modulation ZF TFs are the first class of TFs that bind new open chromatin regions.

### Reprogramming cell fate kinetics and efficiency are improved with hyposmotic modulation

**Figure 5:**
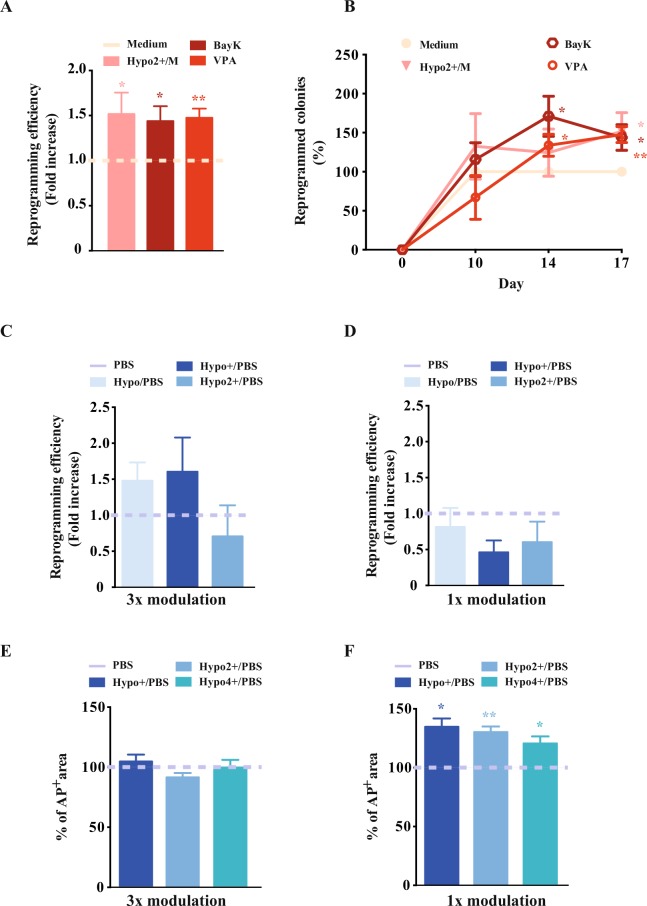
Effect of hyposmotic modulation in cell fate reprogramming. (**A**) and (**B**) Reprogramming efficiency and kinetics, measured as the number of alkaline phosphatase positive colonies, with different modulation protocols in UCB CD34^+^CD133^+^ cells (normalised to the hESC medium condition). Two small molecules (VAP and BayK) with proven activity as reprogramming boosters were used for comparison with hyposmotic condition. Valproic Acid (VPA), is a chromatin modulating small molecule and BayK is a calcium agonist. (**C**) and (**D**) Fold increase in reprogramming with different hyposmotic modulation protocols in UCB CD34^+^CD133^+^ cells (normalised to the PBS condition). (**E**), (**F**) Percentage of alkaline phosphatase positive cell culture area with different hyposmotic modulation reprogramming protocols in NDHF (normalised to the PBS condition). The different osmotic modulation protocols are described in the legend of each graph. The variations presented are the mean value ± SEM (n ≥ 3) and the changes are statistically significant at the time points highlighted in the graphs (*p value < 0.05; **p value < 0.01).

Several strategies, with variable efficiencies, have been proposed in the last years for the generation of iPSCs from different cell sources. There are several reports that even sub-compartments of the same cell source show different reprogramming efficiency (Supplementary Fig. S9B)^42–44^. This is believed to be due to chromatin structure and most reprogramming protocols include the use of inhibitors of chromatin modulating enzymes. These approaches induce an open chromatin state that increases the plasticity of the target cell allowing the access of the reprogramming factors to new genomic locations so that the transcriptional repertoire can be changed and the pluripotent state can be achieved. The fact that several reprogramming cocktails have used different chromatin modulating drugs (CMDs) depending on the cell source used to generate iPSCs indicates that these drugs show some type of bias towards some mechanistic specificity inherent to the CMDs or genomic location^45–49^. There is still no method that can open chromatin irrespective of the molecular players involved, something purely physical that can quickly modulate the players involved in transcription. We devised a method that uses a hyposmotic stimulus that transiently swells the cells and opens the chromatin. With this method we have observed that CD34^+^-UCB cells acquire a pluripotent stem cell phenotype quicker than using regular iPSC-derivation conditions (based on valproic acid (VPA) and BayK a calcium agonist) (Fig. 5A,B). Briefly, the hyposmotic modulation was done during the first week of reprogramming and we have used a lentiviral vector developed by Warlich *et al*., which has a fluorescent reporter protein (dTomato) that denotes the occurrence of epigenetic remodelling within the cell genome^50^ (further details in the Supplementary experimental procedures). Therefore this feature also allowed us to analyse the kinetics of the epigenetic remodelling by analysing the presence of the fluorescent reporter protein, because during active epigenetic remodelling the viral promoter leading the expression of the fluorescent reporter is inactivated. This kinetic profile shows a significant increase of non-reprogrammed colonies in the conditions where small molecules have been used and not in the hypo2+/M condition (Supplementary Fig. S9C). The epigenetic reprogramming when using chromatin modulating drugs happens later probably meaning that these molecules have a long lasting chromatin effect, when compared with the osmotic modulatory condition (Supplementary Fig. S9C).

Hyposmotic modulation impacts in the reprogramming efficiency output, (Fig. 5A–D) and depending on the target population the results are distinct. The same type of modulation used in human dermal fibroblast reprograms more efficiently (with less noise) than the heterogeneous population of UCB cells (Fig. 5E,F).

Next, to complement these proof-of-principle studies, a well-characterised cell fate switch system of transdifferentiation was used. The effect of hyposmotic modulation was tested in a B-cell line with a β-estradiol-inducible form of C/EBPα, where the original cells can be converted into macrophage-like cells with high efficiency^51^. C/EBPα has been described as a pioneer factor and therefore this transdifferentiation system has a high and robust efficiency which can be related to the ability of this TF to bind to DNA even in packed chromatin areas^51,52^. The detailed description of this experiment is provided in the supplementary experimental procedures, but briefly, the experimental layout consisted in the induction of transdifferentiation by the addition of 100 nM of β-estradiol and the transient osmotic modulation (once a day) started one day after this point.

**Figure 6:**
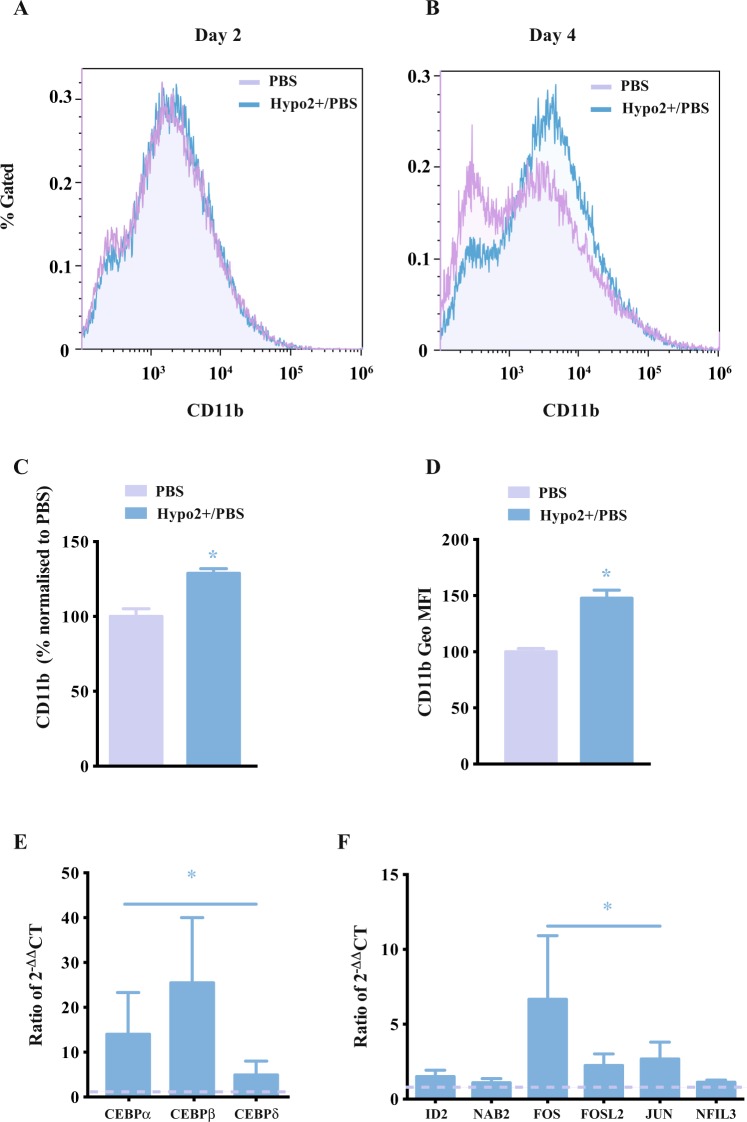
Effect of hyposmotic modulation in cell fate transdifferentiation. (**A**,**B**) CD11b expression by the HAFTL C10 cell line during the transdifferentiation. Although the images provided are from a single experiment, the same experiment was done at least two more times with similar results. (**C**) CD11b expression (% of cells expressing CD11b relative to PBS-control) by the HAFTL C10 cell line at day 4 of transdifferentiation. (**D**) CD11b expression (Geo MFI, geometric mean fluorescence intensity) by the HAFTL C10 cell line at day 4 of transdifferentiation. (**E**) Transcriptional characterization of CEBP-family genes assessed at day 4 of transdifferentiation in HAFTL C10 cell line by qRT-PCR (ratio between the expression levels (2−ΔΔCT) in the two transdifferentiation conditions) (ANOVA test used). (**F**) Gene expression of CEBP-related transcription factors assessed at day 4 of transdifferentiation in HAFTL C10 cell line by qRT-PCR (ratio between the expression levels (2−ΔΔCT) in the two transdifferentiation conditions) (ANOVA test used). The different osmotic modulation protocols are described in the legend of each graph. The data presented corresponds to the mean value ± SEM (n ≥ 3) and the changes are statistically significant at the time points highlighted in the graphs (*p value < 0.05).

The hyposmotic stimulus promotes an increase in the percentage of cells expressing CD11b along the transdifferentiation process, a macrophage-specific marker as shown in Fig. 6. Within the transdifferentiation process, when β-estradiol is added to the cell culture media, the hyposmotic modulation (hypo2+/PBS) has a significant impact in the percentage of cells expressing the macrophage-related marker CD11b at day 4 when compared to the control (Fig. 6C,D) and respective negative controls (Supplementary Fig. S9G).

The hyposmotic modulation has a significant positive effect on the expression of CEBPα during the transdifferentiation but interestingly it also promotes the expression of CEBPβ and CEBPΔ (Fig. 6E,F). Moreover, some of the genes, shown to be increased by a previous independent microarray analysis^51^, were also significantly increased after the transdifferentiation with hyposmotic transient modulation when compared to the control group. These genes included FOS, FOSL2, JUN and NFIL3 (Fig. 6E,F).

## Discussion

Osmotic regulation is very important in several physiological and pathological events. The great majority of studies in the literature have focused on the effect induced by hypertonicity but has shown in this study the hyposmotic modulation provides exciting and underexplored avenues to change chromatin architecture and transcription activity.

Hyposmotic modulation induces numerous cellular changes such as: the increase in cell size, decrease in intracellular levels of ATP, and increase in intracellular calcium. The hyposmotic modulation is a powerful tool that enables the manipulation of two of the most powerful molecules in the cell, ATP and calcium. Fluctuations in these molecules can have a tremendous role in cellular function and phenotype. Particularly, in the stem cell manipulation field, alterations in the calcium signalling can have a great impact in stem cell potency^53^. Also, ATP is not only an important extracellular signalling molecule but is crucial for most intracellular kinetic reactions; the impact of ATP levels on transcription rate observed upon osmotic modulation is in line with previous observations^31^ and may be crucial for some of the transcription changes observed. These may have a profound effect in the splicing repertoire produced by the cell^31,54^ and may impact in stem cell fate decisions^31,55^. This side-effect induced by hyposmotic modulation has to be considered when longer/multiple hyposmotic stimuli are required, and in these scenarios transcription speed can be rescued or modulated by providing ATP and stabilising agents to the cells after hyposmotic modulation. Nevertheless, the fact that the hyposmotic modulation enabled the recruitment of more RNA Pol II into chromatin is a main result that opens avenues of new possibilities to modulate transcription and cell fate. One of such strategies is to introduce high levels of exogenous TFs inside the cell and therefore favour the recruitment of RNA Pol II to the binding sites of these TFs, in excess within the cell nucleus, and promote the transcription of specific target transcripts. To understand the biological meaning of RNA Pol II binding is not a straightforward or easy task, but in this study we show that the hyposmotic modulation promotes a general significant increase of the different forms of RNA Pol II bound to chromatin. We believe that this is a combined effect of chromatin relaxation and recruitment of RNA Pol II by different TFs. The biophysical impact of the entrance of water into the cell might play a crucial role in the nuclear architecture and chromatin structural modifications. Technically it is difficult to prove such theory but some studies have shown that the physical impairment of chromatin structure plays a role in the overall chromatin structure and transcriptional activity^26–28^. For instance, the increase in H4K16 acetylation is synonym of transcriptional activity^31,55–58^. It can be argued that the results obtained with hyposmotic modulation could be achieved by the use of drugs and small molecules that promote chromatin remodelling. But the biophysical strategy here described presents the advantage of a broader action, avoids expensive reagents and can be easily tailored for application in all cellular scenarios.

Changing the water content of the cell may have an impact in the hydration state of the DNA, condition the methylation state of different genomic areas, and also favour the binding of specific TFs. The specific access of TFs to cell’s DNA is a crucial step for fate determination. In this study, we conducted in silico analyses to look for TFs that could be working as “osmotic sensors”. Although we cannot provide at this time experimental validation for most of this *in silico* analyses we would like to highlight that eight out of the top ten TFs, computed by PASTAA, are classified as Zinc Finger TFs. Further studies are needed to assess the importance of this class of TFs in osmotic regulation in mammals but from an evolutionary perspective this makes sense. The osmotic stress response is mediated by ZF proteins in *Arabidopsis* (AtRZF protein), *Festuca arundinacea* (FaZF protein), and the rice plant (ZF protein 36)^59,60^. Therefore, although not very explored in humans, the osmotic modulation response might be also mediated by ZF proteins. From a bioengineering perspective, this ZF-TF effect can hold a huge potential for future application in engineered ZF-protein strategies for gene-editing and differentiation, dedifferentiation and transdifferentiation protocols with various applications in cell therapy and cancer research.

Here we show in a series of proof-of-principle experiments that under specific hyposmotic stimulation it is possible to improve the kinetics and efficiency of transdifferentiation and iPSC-reprogramming protocols. The changes in the efficiency of cell fate modulation are modest but this may be related to the fact that these processes are mainly dependent on pioneer factors which do not need chromatin to be accessible to bind the DNA target sites. This knowledge also opens other possibilities for the hyposmotic modulation as a methodological tool for discovery of new pioneer TFs; that in theory would not have their binding ability impaired by chromatin hyposmotic induced relaxation. Additionally, it could be an interesting tool to unveil characteristics of pioneer (such as PU.1) and non-pioneer TFs that are affected by the hydration state of the DNA.

Therefore, the full power of the impact of hyposmotic modulation on chromatin structure and transcriptional changes might be shadowed by the action of the pioneer factors, which are present in a stoichiometric advantage within the cell nucleus.

Nevertheless, there was a significant impact in the reprogramming efficiency in fibroblasts and we believe that the kinetic changes seen in UCB cell reprogramming also show the ability of the osmotic stimulus to modulate the speed of the process. In the case of the transdifferentiation experiment, CEBPα can also have a pioneer activity^51,52^ and mask the full potential of hyposmotic modulation.

These results show that hyposmotic modulation impacts in the kinetics and efficiency of cell fate alteration protocols but there is certainly room for further developments. To unveil the full potential of the hyposmotic modulation would be very interesting to use models where cell fate changes are especially hard, like the mature B-cell reprogramming scenario. In this context, a pulse of CEBPα expression followed by induction of Yamanaka factors expression reached a high fold increase (>100) in the reprogramming efficiency^61^. We believe that in this challenging system it would be possible to understand if the CEBPα pulse has a role in chromatin opening, to allow the access of the pluripotency-related TFs, and if this effect could be mimicked by an hyposmotic transient modulation scheme.

To use hyposmotic modulation as a cellular modulation tool, several variables need to be considered such as the cell type, and particularly, the time and intensity of the stimulus need to be specifically tailored to each cell type. The susceptibility of the different cell types to different hyposmotic modulation schemes is also highly dependent on biophysical characteristics. Cells in suspension or adherent to cell culture substrates have different cytoskeleton structures and therefore respond differently to increased water content and cell size changes^62^. Another aspect that probably influences the process is the previous contact with specific extracellular matrix components. In this case the memory of the physical characteristics of the environment can alter the efficiency or the kinetics of the process.

Future work with water homoeostasis and the ionic balance will certainly provide fruitful knowledge for the understanding of normal and pathological cell behaviour in specific contexts. In our opinion, osmotic modulation provides a cheap, easy and scalable strategy that withholds a huge potential for future development of targeted therapeutic approaches either by the impact on cellular function, cellular fate determination, and metabolism but especially by its ability to influence transcription.

## Materials and Methods

### Cell collection and culture

Cord-blood cells were collected from donors after signed informed consent and ethical approval was granted by the ethics committee of *Hospital Infante D*. *Pedro Aveiro* in Portugal. All experiments were performed in accordance with relevant, approved guidelines and regulations. The experimental protocols were also approved by the ethics committee of *Hospital Infante D*. *Pedro Aveiro* in Portugal. Several cell lines were used and maintained at 37 °C in humidified incubators with 5% CO2 in specific growth culture conditions as further described within the supplementary experimental procedures.

### Osmotic modulation

The cells were exposed to different osmotic conditions for different times at 37 °C with 5% CO2. In Supplementary Table S1 the specific conditions used are described.

### Cell imaging and cytometry

For cell imaging, a confocal LSM 710 (Carl Zeiss) and a high content imaging platform IN Cell 2200 Analyzer (GE Healthcare) were used. For analysis of the images from fluorescence and confocal microscopy, ImageJ and ZEN software were used. For a specific analysis of mitochondrial morphology a specific plugin was used in ImageJ and references for this tool are described in the results. For IN Cell 2200 Analyzer image data analysis, the equipment software was used.

For cell cytometry, GalliosTM (Beckman Coulter), BD FACSVerse (Becton Dickinson Biosciences), BD FACSARIA II (Becton Dickinson Biosciences) and BD Accuri C6 (Becton Dickinson Biosciences) flow cytometry machines were used. For analysis of the acquired data, Kaluza Analysis 1.5, and FlowJo software were used.

### RNA Pol II transcription analyses

To assess transcription, Click-iT RNA Alexa Fluor 488 Imaging Kit (Molecular Probes) was used as per the manufacturer’s protocol. Flow cytometry was used to assess the fluorescent signal (GalliosTM). Different time points for 5-Ethynyl Uridine (EU) incorporation were evaluated.

The number of active molecules of RNA polymerase II (RNA Pol II) was also measured after “run on” experiments using 5-Ethynyl-UTP (EUTP) as detailed supplementary experimental procedures.

### Enzymatic digestions

DNase I assay and MNase test were performed in 5 × 10^5^ K562 nuclei and the digestions were carried out as further explored in the supplementary experimental procedures.

### Chromatin immunoprecipitation Sequencing

K562 cells were grown in complete growth medium and after osmotic modulation the cross-link was done with formaldehyde. Chromatin immunoprecipitation was carried out using antibodies against RNA Pol II different forms.

### Fluorescence Loss in Photobleaching – FLIP

Briefly, in FLIP experiments a nuclear area is continuously photobleached (Fig. 3A), and the fluorescence intensity is a measure of the amount of RNA Pol II-GFP molecules in the unbleached area. The decay in fluorescence is then due to the free RNA Pol II-GFP molecules entering the bleached area. Different dynamic regimes in the fluorescence decay thus indicate the presence of different dissociation kinetics (Hieda *et al*.^32^). The simple kinetic model shown in Supplementary Fig. S5 can be used to estimate the reaction rate constants. Further details are provided in Supporting info.

### Lentiviral production

The viral packaging was performed in 293 T with the appropriate amounts of transfection agent Lipofectamine 2000 and plasmids of interest with a third generation lentiviral vector system^63^.

### Reprogramming protocol

The cell reprogramming protocol used was based on the report in^43^ and is detailed in supplementary experimental procedures. Briefly, after viral transduction with the reprogramming TFs, the hyposmotic modulation schemes were done in the first week of reprogramming and the assessment of alkaline positive colonies was done 2 weeks after viral infection.

### Transdifferentiation protocol

The transdifferentiation protocol used was based on^51^, whom kindly donated the HAFTL-C10 cell line. The protocol used is detailed in the supplementary experimental procedures. Briefly the transdifferentiation was induced by addition of 100 nM of β-estradiol and the osmotic modulation was done one day after the induction of transdifferentiation (daily transient hyposmotic modulation until the end point day).

All data generated or analyzed during this study are included in this manuscript and its Supplementary Information files.

## Electronic supplementary material

Supplementary Information

Supplementary Dataset 1
