# Author Correction: Osmotic modulation of chromatin impacts on efficiency and kinetics of cell fate modulation

**DOI:** 10.1038/s41598-018-29328-3

**Published:** 2018-07-23

**Authors:** A. F. Lima, G. May, J. Díaz-Colunga, S. Pedreiro, A. Paiva, L. Ferreira, T. Enver, F. J. Iborra, R. Pires das Neves

**Affiliations:** 1 UC-Biotech, CNC - Center for Neuroscience and Cell Biology, University of Coimbra, 3060-197 Cantanhede, Portugal; 2 Faculty of Science and Technology, University Nova of Lisbon (MIT-Portugal PhD Program), 2829-516 Caparica, Portugal; 3 University College London, Gower Street, London, WC1E 6BT UK; 4 Centro Nacional de Biotecnología, CSIC. Darwin 3, Campus de Cantoblanco, 28049 Madrid, Spain; 5 Unidade de Gestão Operacional de Citometria, Centro Hospitalar e Universitário de Coimbra, 3000-075 Coimbra, Portugal; 6 Faculty of Medicine, University of Coimbra, 3004-504 Coimbra, Portugal; 7 Institute for Interdisciplinary Research, University of Coimbra, 3030-789 Coimbra, Portugal; 8 Coimbra Institute for Clinical and Biomedical Research, Faculty of Medicine,University of Coimbra, 3004-504 Coimbra, Portugal

Correction to: *Scientific Reports* 10.1038/s41598-018-25517-2, published online 08 May 2018

The original version of this Article contained a typographical error in the spelling of the author J. Díaz-Colunga, which was incorrectly given as J. Colunga.

As a result, the sentence in the Author Contributions section,

“J.C. analysed ChIP-Seq data”

now reads

“J.D.-C. analysed ChIP-Seq data”

These errors have now been corrected in the PDF and HTML versions of the Article, and in the accompanying Supplementary Information.

