# Supplementary material for: Osmotic modulation of chromatin impacts on efficiency and kinetics of cell fate modulation
Source: Sci Rep. 2018 May 8;8:7210. doi: 10.1038/s41598-018-25517-2 (PMC5940679; doi:10.1038/s41598-018-25517-2)
Supplement: Supplementary file 1 — Supplementary Information [file 41598_2018_25517_MOESM1_ESM.docx]

**Supplementary Information**

**Osmotic modulation of chromatin impacts on efficiency and kinetics of cell fate modulation**

Lima, A.F.^1,2^, May G.^3^, Díaz-Colunga J.^4^, Pedreiro, S.^5^, Paiva, A. ^5^, Ferreira, L.^1,6^, Enver T.^3^, Iborra F.J. ^4^, Pires das Neves, R.^1,7*^

^1^ UC-Biotech, CNC - Center for Neuroscience and Cell Biology, University of Coimbra, 3060-197 Cantanhede, Portugal; ^2^ Faculty of Science and Technology, University Nova of Lisbon (MIT-Portugal PhD Program), 2829-516 Caparica, Portugal; ^3^ University College London, Gower Street, London, WC1E 6BT, UK; ^4^ Centro Nacional de Biotecnología, CSIC. Darwin 3, Campus de Cantoblanco, 28049 Madrid, Spain; ^5^ Centro Hospitalar e Universitário de Coimbra 3000-075 Coimbra, Portugal; ^6^ Faculty of Medicine, University of Coimbra, 3004-504 Coimbra, Portugal; ^7^ Institute for Interdisciplinary Research, University of Coimbra 3030-789 Coimbra.

* Corresponding author: Ricardo Pires das Neves ([ricardo.neves@uc-biotech.pt](mailto:ricardo.neves@uc-biotech.pt))

- **Supplementary tables**
  - Supplementary Table S1

Table S1 – Summary of the osmotic conditions used to modulate the cellular environment.

The abbreviated nomenclature presented in this table is used throughout the paper to represent the conditions described here (in the last column min stands for minutes). The complete growth medium osmolality was assumed to be within the isosmolar range (≈280mOsm/Kg) and the PBS osmolality was the average osmolality range provided by the supplier (280 - 315 mOsm/kg).

| **Name given to the experimental condition** | | **Medium/**  **solution used** | **Distilled water in the total volume of the cocktail** | **NaCl % in the total volume of the cocktail** | **Type of stimuli** |
| --- | --- | --- | --- | --- | --- |
| Hypo/M | Hyposmotic Media | Complete growth culture medium (≈280mOsm/Kg) | 1/4 (≈210mOsm/Kg) |  | Permanent or transient (15 min) in the osmotic modulation cocktail during the experiment at 37ºC, 5% CO_2_. |
| Hypo+/M |  |  | 1/3 (≈187mOsm/Kg) |  |  |
| Hypo2+/M |  |  | 1/2 (140≈mOsm/Kg) |  |  |
| Hypo3+/M |  |  | 2/3 (93≈mOsm/Kg) |  |  |
| Hypo4+/M |  |  | 3/4 (≈70mOsm/Kg) |  |  |
| Hyper/M | Hypertonic Media |  |  | 1.8  (≈560mOsm/Kg)* |  |
| Hypo/PBS | Hyposmotic solutions | Phosphate-Buffered Saline (PBS) (≈297.5mOsm/Kg) | 1/4 (≈223mOsm/Kg) |  | Permanent or transient (15 min) in the osmotic modulation cocktail during the experiment at 37ºC, 5% CO_2_ |
| Hypo+/PBS |  |  | 1/3 (≈198mOsm/Kg) |  |  |
| Hypo2+/PBS |  |  | 1/2 (≈149mOsm/Kg) |  |  |
| Hypo4+/PBS |  |  | 3/4 (≈74mOsm/Kg) |  |  |
| Hyper/PBS | Hypertonic solution |  |  | 1.8  (≈600mOsm/Kg)* |  |

- - Supplementary Table S2 (Please see Supplementary Dataset File)

Table S2 – Top genes in ChIP-Sequencing “new peaks” gene list. These peaks are only present in the hypo2+/PBS condition when compared to the correspondent PBS condition for the different chromatin immunoprecipitations performed (RNA Pol II Total, RNA Pol II PhosphoSer5 and RNA Pol II PhosphoSer2). The presented genes are in descending fold enrichment and both name and generic function of the genes is given. The gene name and general function were obtained and adapted from the human gene database (http://www.genecards.org/).

Supplementary Figures

- - Supplementary Figure S1

**

**

Fig. S1 – Impact of different osmotic environments in K562 cell physiological parameters. (A) Effect of constant osmotic modulation on cell size in K562 cells.

Forward side scatter percentage (cell size %) mean value variation (± SEM) over time (n=2**).** (B) Effect of constant osmotic modulation on ROS levels in K562 cells.

Cellular ROS (% of fluorescence signal normalized to time 0h) levels (±SEM) over time (n≥3). (C) Effect of constant osmotic modulation on mitochondrial superoxide levels in K562 cells.

Mitochondrial superoxide (% of fluorescence signal normalized to time 0h) changes (±SEM) over time (n≥3). (D) Effect of constant osmotic modulation on mitochondrial membrane potential (ΔΨm) in K562 cells. Mitochondrial membrane potential (% of fluorescence signal normalized to time 0h) differences (±SEM) over time (n=3). (E) Effect of constant osmotic modulation on intracellular free calcium levels in K562 cells. Intracellular free calcium level (% of fluorescence signal normalized to time 0h) changes (±SEM) over time (n=3). (F) Effect of transient osmotic modulation on intracellular free calcium levels in K562 cells. Intracellular free calcium level (% of fluorescence signal normalized to control) changes (±SEM) over time in K562 cells (n=4) after a transient modulation (15 min). For all the graphs the different osmotic modulation protocols are described in the legend. These changes are statistically significant at the time points highlighted (*p value < 0.05;** p value < 0.01; *** p value < 0.001).

- - Supplementary Figure S2

**

**

Fig. S2 – Effect of CPDA-1 induced ionic changes in UCB-MNCs phenotype. (A) CPDA-1 influence in intracellular calcium levels in UCB-MNCs.

Intracellular free calcium levels were assessed with the eFluor514 probe by flow cytometry (BD FACSCalibur) and the values of the average intensity (± SEM) were normalized to the control group (named 0 in the graph) (n=2). (B) CPDA-1 influence on the total UCB-MNCs number.

The cell number was achieved by manual counting with a Neubauer chamber right after the 48h exposure of UCB-MNCs to CPDA-1. The average cell numbers presented (± SEM) were normalized to the control group (n=3). (C) CPDA-1 influence in total UCB-MNCs number.

The cell number was achieved by manual counting with a Neubauer chamber right after the 4 days of standard UCB-MNCs culture. The average cell numbers presented (± SEM) were normalized to the control group (n=3). (D) CPDA-1 influence in the expression of CD34 within UCB-MNCs.

After 4 days of standard UCB-MNC culture, the evaluation of expression of CD34 marker was done by flow cytometry (BD Accuri C6). The percentage of UCB-MNCs expressing only CD34 was calculated, using FlowJo cytometry analysis software, and the average percentage of cells presented (± SEM) were normalized to the control group (n=3). (E), (F) CPDA-1 influence in the expression of CD34 and CD133 within UCB-MNCs.

After 4 days of standard UCB-MNCs culture, the evaluation of expression of CD133 (E) or CD34 and CD133 (F) markers was done by flow cytometry (BD Accuri C6). The percentage of UCB-MNCs expressing both these markers was calculated, using FlowJo cytometry analysis software, and the average percentage of cells presented (±SEM) were normalized to the control group (n=3). For all the graphs the different protocols used are described in the legend. These changes are statistically significant at the time points highlighted (*p value < 0.05;** p value < 0.01; **** p value < 0.0001).

- - Supplementary Figure S3





Fig. S3 – Impact of different osmotic environments in normal dermal human fibroblasts (NDHF) cell physiological parameters. (A) Effect of transient osmotic modulation (15 minutes) on intracellular ATP levels (% normalized to the medium/PBS condition) (±SEM; n=3). (B) Effect of transient osmotic modulation (15 minutes) on extracellular ATP levels (% normalized to the medium/PBS condition) (±SEM; n=3). (C) Effect of constant osmotic modulation on mitochondrial membrane potential (ΔΨm) in NDHF m Mitochondrial membrane potential (% of fluorescence integrated intensity normalized to the control) differences (±SEM) over time (n=3). (D) Effect of constant osmotic modulation on intracellular free calcium levels in NDHF cells. Intracellular free calcium level (% of fluorescence integrated intensity normalized to the control) changes (±SEM) over time (n=3)**.** (E) Effect of transient osmotic modulation on mitochondrial membrane potential (ΔΨm) in NDHF cells. Mitochondrial membrane potential (% of fluorescence integrated intensity normalized to the control) differences (±SEM) over time in NDHF cells (n=3) after a transient modulation (15 min). (F) Effect of transient osmotic modulation on intracellular free calcium levels in NDHF cells. Intracellular free calcium level (% of fluorescence signal normalized to control) changes (±SEM) over time in NDHF cells (n=3) after a transient modulation (15 min). (G) Effect of constant osmotic modulation on ATP levels (% normalized to medium condition). (H) Morphological changes of eFluor514 stained NDHF after 60min in hypo2+/PBS (scale bar 100μm). For the analysis in (C) to (F**) c**onfocal imaging was used for imaging the stained cells over time (at least 100 cells per condition were analysed). For all the graphs the different osmotic modulation protocols are described in the legend. These changes are statistically significant at the time points highlighted (*p value < 0.05;** p value < 0.01; *** p value < 0.001; **** p value < 0.0001).

- - Supplementary Figure S4

**

**

Fig. S4 – Osmotic influence in nuclear structures. (A) Confocal imaging with DAPI stained K562 cells, previously presented to different osmotic solutions.

The cells were fixed and imaged straight after exposure to different osmotic modulation conditions, shown in the figure. Scale bar = 10μm. (B) Confocal imaging with DAPI stained UCB-MNCs, previously presented to different osmotic solutions.

The cells were fixed and imaged straight after exposure to different osmotic modulation conditions, shown in the figure (scale bar = 2μm**)** and respective coefficient of variance of the DAPI staining (approximately 100 cells per condition were analysed). (C) Effect of transient hyposmotic modulation on cell nuclear area in HeLa cells.

Nuclear area normalized to control (100%) over time. (D) Effect of transient hyposmotic modulation on the nuclear organization in HeLa cells.

Nuclear DAPI intensity normalized to control **(100%)** over time. (E) Effect of transient hyposmotic modulation on cell nuclear area in HeLa cells.

Nuclear area normalized to control (100%) over time**.** (F) Effect of transient hyposmotic modulation on cell nuclear organization in HeLa cells.

Nuclear DAPI intensity normalized to the control (100%) over time. For all the graphs statistically significant differences in comparison with the respective control are highlighted (*p value < 0.05; **p value < 0.01; *** p value < 0.001). The osmotic modulatory conditions used, are shown in each legend.

- - Supplementary Figure S5


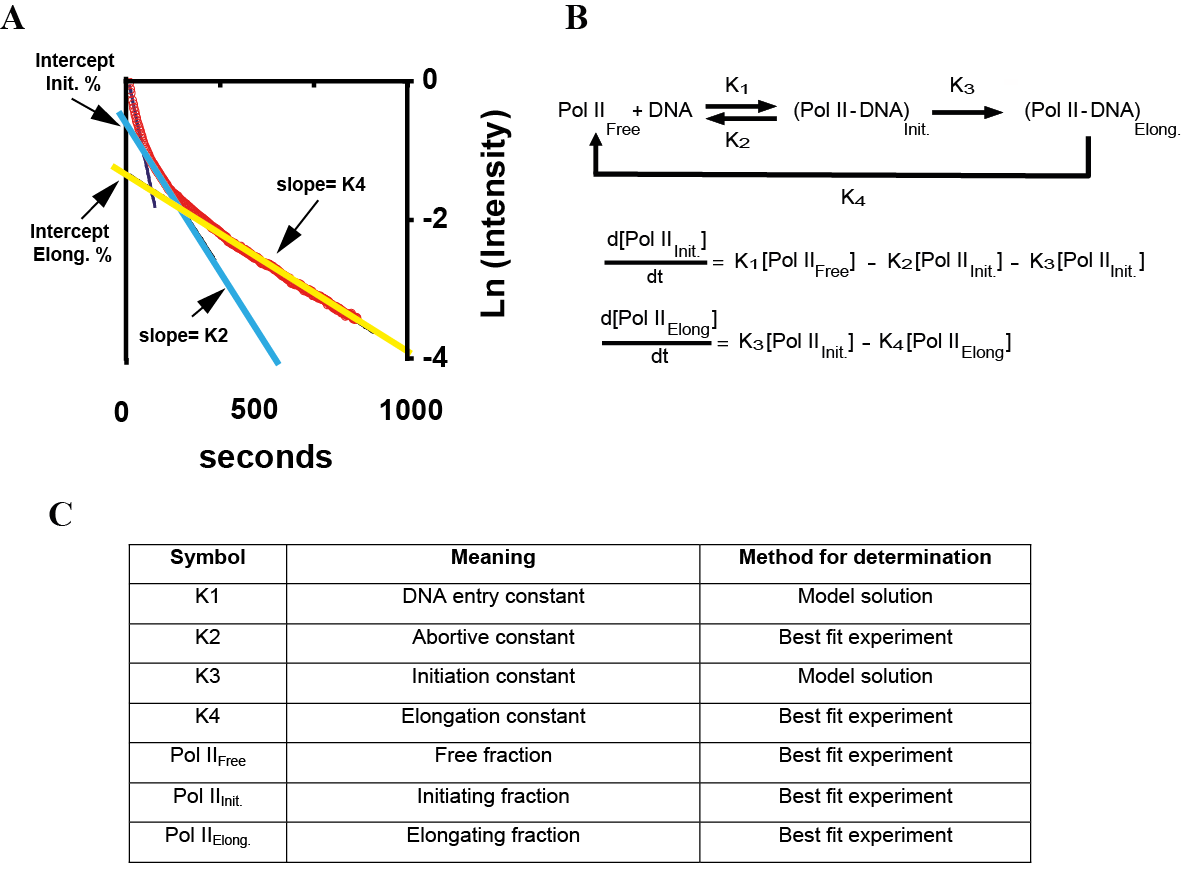


**Fig. S5 – FLIP, Fluorescence Loss in Photobleaching analyses.** (A) Fluorescence decay curves were analysed using Sigma Plot 11.0 for Windows and data were fitted to three populations with an exponential decay ($f=a\times\exp\left( -b\times x \right)+c\times\exp\left( d\times x \right)+g*exp(-h\times x)$; R2>0.99): one free form, one bound to DNA but not fully engaged, and another fully engaged in transcription. (B) FLIP analysis allows to estimate the dissociation constants of the different populations. In order to associate these constants to a functional process, we treat cells with inhibitors of specific steps of the transcription cycle, and then see which kinetic regime is affected. In this way, we can link a decay constant to a specific transcription step ([Hieda et al. 2005](#_ENREF_24), [Sugaya et al. 2000](#_ENREF_54)). (C) For the fitting to the exponential decays, we allowed the three components to optimize with no restriction (always r2>0.99). These fittings allow us to estimate the fraction of the different populations and the rate constants K2 and K4. To obtain the other kinetic constants (K1 and K3) we fitted our data to the steady-state solution of the kinetic model.

- - Supplementary Figure S6

Fig. S6 – Osmotic influence in RNA production, chromatin structure, and histone modification pattern. (A), (B) Impact of different osmolarity in the production of nascent RNA in a HeLa cell line expressing the histone H2B fused to GFP. (B) Quantification of brominated nascent RNA presented in (A) in function of chromatin state (H2B/GFP SD/mean). (C) DNA fragmentation pattern after MNase digestion using K562 cells. The digestion was performed with nuclei isolated from K562 cells that were transiently in different osmotic conditions and with increasing MNase concentrations (0; 12.5; 25; 50; 150; 250; 500 gel units). DNA loading in each lane was 500ng. The osmotic modulatory conditions used are shown in the figure. (D) DNA fragmentation pattern after digestion with DNase I using K562 cells.

The digestion was performed with isolated nuclei from K562 cells which were transiently in different hyposmotic conditions and increasing DNase I concentrations (80; 120; 240; 480; 960; 1960; 2400 U/mL). The DNA loading in each lane was 500ng. (E) Representative western blot for protein expression of transcription related elements. The osmotic modulatory conditions used are shown in each legend (the diffrerent histone blot was cropped from different gels using the same exposure conditions and loading control). (F**)** Immunolabeling quantification of the fluorescence signal of H4K16 acetylation in UCB-MNCs presented to different osmolarity conditions.

Straight after exposing UCB-MNCs to different osmotic modulations cocktails (n=2) the cells were fixed and the immunolabeling was performed and the mean intensity fluorescence (±SEM) evaluated with the high content imaging platform IN Cell Analyzer 2200. (G) Immunolabeling quantification of the fluorescence signal of H4K16 acetylation HeLa cells after a transient hyposmotic modulation.

Straight after exposing HeLa cells to different osmotic modulations cocktails (n=3) the cells were fixed and the immunolabeling was performed. The mean intensity fluorescence (±SEM) normalized to the time point 0h was evaluated with the fluorescence microscope Axioivert 200M (Carl Zeiss). For all the graphs the osmotic modulatory conditions used are shown in each legend. Statistically, significant differences in comparison with the timepoint 0h are highlighted in the graph (**p value < 0.01).

- - Supplementary Figure S7

Fig. S7 – Reliability check of the Chip-Seq dataset. Bioinformatic analyses to check the integrity and degree of agreement between Chip-Seq peaks obtained from pull-downs using with different antibodies for RNA pol II. Venn diagrams showing the overlap between feature IDs of total (green) and initiating (red) polymerase ChIP-seq peaks. (A) Control sample at 0h (overlap ~87%). (B) Control sample at 1h (overlap ~84%). (C) Hypo sample at 0h (overlap ~94%). (D) Hypo sample at 1h (overlap ~85%).

- - Supplementary Figure S8

**

**

Fig. S8 – Effect of osmolarity in the RNA Pol II chromatin binding profile. (A) Effect of transient osmotic modulation on DNA recovery after chromatin immunoprecipitation.

The DNA recovered (normalized %) in each condition was normalized to the initial amount of DNA (±SEM; n=2) present within each control before immunoprecipitation (input). (B) The ratio of DNA amount recovered after chromatin immunoprecipitation.

The ratio here presented is between the normalized DNA amounts (±SEM; n=2) recovered after ChIP for the isosmotic sample versus the hyposmotic modulated sample$\left( \frac{\mathbf{PBS}}{\mathbf{hypo2+/PBS}} \right)$. (C) Ranked prediction of transcription factors involved in the regulation of the “new peaks” gene list of ChIP-Seq data for RNA Pol II PhosphoS5 at time-point 0h and 1h.

Immediately after the hyposmotic stimulus (0h) eight out of the ten higher ranked transcription factors belong to the superclass of “zinc-coordinating DNA-binding domains”. After 1 hour this is no longer the case and only 3 ZF-TFs are listed in the top-10. This analysis was done with PASTAA and classification of TFs was done at http://tfclass.bioinf.med.uni-goettingen.de/tfclass**.** (D) Mitochondrial morphology within different osmolarity environments.

MitoGreen HeLa cells were imaged on an LSM 710 (Carl Zeiss) confocal microscope (scale bar 20μm). The acquisition time and osmolarity environment used is shown in the figure. (E) Mitochondrial fragmentation index (f-index) within different osmolarity environments.

MitoGreen HeLa cells were imaged on an LSM 710 (Carl Zeiss) confocal microscope. The analysis of the volume of fragments was done using an ImageJ plugin (for further details see supplementary experimental procedures in S1_Text) and the f-index (±SEM) was calculated afterwards. For all the graphs the osmotic modulatory conditions used are shown in each legend. Statistically, significant differences in comparison with the timepoint 0h are highlighted in the graph (* p value<0.5; ***p value < 0.001).

- - Supplementary Figure S9

**

**

**Fig. S9 – Effect of osmolarity in cell fate reprogramming models.** (A) Osmotic modulation doesn’t have significant impact on viability (ATP levels) of UCB-MNC cells. (B) Reprogramming efficiency differences (± SEM) between UCB isolated cells CD34+CD15+ versus CD34+CD15- (n=2). The fold increase in reprogramming efficiency is normalized to the CD34+CD15+ condition (considered to be equal to one). (C) Kinetics of non-reprogrammed colonies with different modulation protocols in UCB CD34+CD133+ cells. The “non-reprogramming” kinetics was assessed by counting the number of colonies that did express the lentiviral fluorescent reporter protein (non-reprogrammed colonies) at days 10, 14 and 17. The control condition was considered to be 100% and the other conditions were normalised to this value. The results of hyposmotic modulation on UCB cell “non-reprogramming” over time and the chromatin modulating drugs are shown (±SEM; n=3). (D) Immunostaining against Nanog and DAPI staining of UCB-MNCs derived iPSCs (scale bar 100μm). (E) NDHF cell morphology after 15min in hypo2+/PBS (scale bar 100μm). Image acquired in IN Cell 2200 Analyzer (GE Healthcare). (F) NDHF cell morphology after 15min in hypo4+/PBS (scale bar 100μm). Image acquired in IN Cell 2200 Analyzer (GE Healthcare). (G) HAFTL C10 cell line phenotypic characterization assessed during the transdifferentiation process and respective negative controls. HAFTL C10 cell line flow cytometry analysis of CD11b expression during cell transdifferentiation within the control condition (PBS) and the transdifferentiation condition with osmotic modulation (hypo2+/PBS) and respective negative controls. Although the images provided are from a single experiment, the same experiment was done at least two more times with similar results. For all the graphs the change is statistically significant when highlighted (*p value < 0.05; ***p value<0.001).

- **Supplementary Experimental Procedures**

**Cell culture**

Several cell lines were used and maintained at 37ºC in humidified incubators with 5% CO2 in specific growth culture conditions as described below.

A chronic myelogenous leukaemia cell line (K562 – ATCC CCL-243) was kept in Roswell Park Memorial Institute (RPMI) medium supplemented with 10% heat-inactivated foetal bovine serum (hiFBS) and 100units/mL of penicillin and 100µg/mL of streptomycin.

Normal dermal human fibroblasts (NDHFs – ATCC PCS-201-010) were maintained in Dulbecco's Modified Eagle Medium (DMEM) supplemented with 10% foetal bovine serum (FBS) and 100units/mL of penicillin and 100µg/mL of streptomycin.

The human adenocarcinoma cell line (HeLa – ATCC CCL-2) was maintained in DMEM supplemented with 10% FBS and 100units/mL of penicillin and 100µg/mL of streptomycin.

The human embryonic kidney cell line (293T – ATCC CRL-3216) was grown in RPMI supplemented with 10% FBS and 100units/mL of penicillin and 100µg/mL of streptomycin.

For reprogramming experiments a feeder layer was prepared from mouse embryonic fibroblasts (MEFs – GlobalStem GSC-6001). Before inactivation, MEFs were expanded and grown in DMEM supplemented with 10% FBS and 100units/mL of penicillin and 100µg/mL of streptomycin. After 2 passages, the MEFs were inactivated with mitomycin C (8μg/ mL) for 2 hours at 37ºC (MEFsI). The reprogrammed cells were cultured in KnockOut DMEM (KO-DMEM), 20% KnockOut serum replacement (KO-SR), 1mM glutamine, 0.1 mM β-mercaptoethanol, 4 ng/ml of fibroblast growth factor-basic (β-FGF), 1% of non-essential amino acids and 100units/mL of penicillin and 100µg/mL of streptomycin ^1^.

Umbilical cord blood-derived cells were a culture in StemSpan medium supplemented with 100ng/mL of stem cell factor (SCF), fms-related tyrosine kinase 3 ligand (Flt-3L) and 100units/mL of penicillin and 100µg/mL of streptomycin.

An altered Ha-ras-oncogene-transformed mouse cell line (HAFTL-C10) stably expressing a CEBPαER was kindly donated by Professor Thomas Graf. It was maintained in RPMI without phenol red supplemented with 10% FBS (charcoal stripped), 100units/mL of penicillin and 100µg/mL of streptomycin, 50 μM β-mercaptoethanol ^2^. To induce transdifferentiation, these cells were presented to the same culture medium supplemented with 100 nM of β-estradiol, 10 ng/mL of interleukin-3 (IL-3) and colony stimulating factor-1 (CSF-1). Control cells were treated with 0.1% ethanol (solvent of β-estradiol).

The human adenocarcinoma cell line (HeLa – ATCC CCL-2) was modified with a lentiviral vector with an enhanced green fluorescent protein (EGFP) protein targeted to the mitochondrial matrix (kindly provided by Professor Rajeev Gupta and based on a previous construct ^3^) and selected for a stable clone. These cells will be designated by MitoGreen HeLa. This cell line was maintained in DMEM supplemented with 10% FBS and 100units/mL of penicillin and 100µg/mL of streptomycin.

A modified Chinese hamster ovarian (CHO-K1) cell line ^4^ that at 39ºC expresses an RNA Polymerase II-EGFP (RNA Pol II-EGFP) was maintained in DMEM/F12 supplemented with 10% FBS and 100units/mL of penicillin and 100µg/mL of streptomycin. Briefly, this cell line was originated from a mutant CHO-K1 cell line ^5^ with a temperature-sensitive mutation in the largest catalytic subunit of RNA pol II (tsTM4 cell clone). A human RNA Pol II wild-type subunit (hRPB1) was tagged with EGFP and expressed in the tsTM4 cell clone. This construct complemented the defect at the restrictive temperature and enabled the mutant cells to grow normally in contrast to what was observed in the parental cell line ^4^. Under culture conditions at 39ºC the RNA Pol II-EGFP is the main source of transcriptional elongation rather than the endogenous enzyme ^4^.

**Osmotic modulation**

The cells were exposed to different osmotic conditions for different times at 37°C with 5% CO2. The conditions used are described in Supplementary Table S1.

**Staining with probes**

Cell staining with the probes like Carboxy-DCFDA, Click-iT RNA Alexa Fluor 488 Imaging Kit, DiBAC4(3), eFluor 514, MitoProbe DiIC1(5), MitoSOX Red and TMRM was done according to the manufacturer’s instructions. All the fluorescence probes labelling were performed in PBS to avoid non-specific interactions during the staining procedure.

**Immunolabeling**

A standard immunodetection procedure was used. Cells were fixed in 4% paraformaldehyde (EM grade 15710-S, EMS) in PBS for 10 min, washed two times in PBS for 5 min each and permeabilised in 1% TritonX-100 (Sigma T8787) in PBS for 10 min. Cells were then washed twice in PBS for 5 min after which they were immersed in blocking solution, composed of 2% Bovine Serum Albumin (BSA (Sigma A9647)) and 2% FBS (BioSera-S1810) in PBS, for 30 minutes. Cells were then incubated by inversion of the coverslip in a 50 μl drop containing primary antibodies in blocking solution over a parafilm strip, for 1 hour at room temperature (RT). After three washing steps in PBS (10 min each) cells were incubated with the secondary antibodies in blocking solution for another hour at RT. After three washes cells were fixed again in 4% paraformaldehyde for 5 min to crosslink antibodies and antigens and coverslips were mounted and preserved in Vectashield for imaging.

**Antibodies**

The antibodies used throughout the work here described are summarized below:

| Specific target | Application | Catalogue number | Supplier |
| --- | --- | --- | --- |
| Mouse anti-IdU/BrdU | Detection of Br-RNA |  | Caltag |
| Alexa Fluor 488 goat anti–mouse IgG | Detection of Br-RNA |  | Jackson ImmunoResearch |
| donkey anti-mouse IgG | Detection of Br-RNA |  | Jackson ImmunoResearch |
| donkey anti-rabbit IgG tagged with Cy3 | Detection of Br-RNA |  | Jackson ImmunoResearch |
| Normal rabbit IgG | ChIP | 12-370 | Millipore |
| RNA polymerase II CTD repeat YSPTSPS | ChIP | ab817 | Abcam |
| RNA polymerase II CTD repeat YSPTSPS phosphoserine 2 | ChIP | ab5095 | Abcam |
| RNA polymerase II CTD repeat YSPTSPS phosphoserine 2 (H5) | ChIP | ab24758 | Abcam |
| RNA polymerase II CTD repeat YSPTSPS phosphoserine 5 | ChIP | ab5131 | Abcam |
| APC anti-human CD133 | MACS | 130-098-829 | Miltenyi |

**RNA Pol II transcription analyses**

To assess transcription, Click-iT RNA Alexa Fluor 488 Imaging Kit (Molecular Probes) was used as per the manufacturer’s protocol. Flow cytometry was used to assess the fluorescent signal (Gallios). Different time points for 5-Ethynyl Uridine (EU) incorporation were evaluated.

The number of active molecules of RNA polymerase II (RNA Pol II) was also measured after “run on” experiments using 5-Ethynyl-UTP (EUTP). Cells were permeabilised with the physiological buffer with Ficoll and saponin (PBF+ saponin) at 4ºC ^6–8^. After five washes in PBF (in order to wash all the internal pools of nucleotides and other intracellular molecules that may interfere with the transcriptional process), cells were stimulated to transcribe at room temperature in the run on cocktail for 15, 30, 60, 90 and 120 min. Cells were then fixed with 4% PFA for 15 minutes at room temperature. The incorporation of EUTP was assessed with Click-iT RNA Alexa Fluor 488 Imaging Kit (Molecular Probes) according to manufacturer’s protocol and using flow cytometry (Gallios).

BrUTP incorporation into the RNA was done in a similar way to EUTP by permeabilization and washing the internal moiety and after run-on in the presence of appropriate nucleotide cocktail, detection of primary transcripts was done with an antibody anti-IdU/BrdU (5 mg/ml; Caltag Laboratories) that has cross-reactivity for Br-RNA.

..

**Enzymatic digestions**

For the deoxyribonuclease I (DNase I), one unit is defined as the enzyme activity that causes an increase in the absorbance of 0.001 per minute under assay conditions. On the hand, one gel unit of micrococcal nuclease (MNase) is defined as the amount of enzyme required to digest 1 µg of lambda genomic DNA in 15 minutes at 37°C, to the extent that the accumulation of low molecular DNA fragments (100-400 base pairs) disappears on a 1.2% agarose gel. A gel unit corresponds to 0.1 Kunitz units (being a Kunitz unit defined as the amount of enzyme required to release acid-soluble oligonucleotides that produce an absorbance increase of optic density 1.0 at 260 nm in 30 minutes at 37°C).

DNase I assay and MNase test were performed in K562 nuclei extracted with a lysis buffer. The nuclear release was confirmed by staining with diluted trypan blue (1:10 in PBS). For each digestion, 5x105 nuclei were used.

In the case of DNAse, nuclei were resuspended in DNase digestion buffer for 3 minutes at 37ºC. Afterwards, the STOP buffer was added and incubated at 55ºC overnight. For the MNase digestion, the nuclei were resuspended in permeabilization buffer 2 and the amount of MNAse needed was added to each condition. The digestion was performed at 21ºC for 5 minutes and afterwards, the digested nuclei were resuspended in equal amounts of TNESK 2x and lysis dilution buffer. This solution was incubated at 37ºC overnight.

Afterwards, ribonuclease A (RNase A) was added to the mixture and incubated for 30 minutes at 37ºC. The digests were purified for DNA content (as mentioned in the section for DNA and RNA extraction). DNA was quantified with Nanodrop2000 (Thermo Scientific) and 500ng of DNA were run in TAE buffer in a 1% agarose gel for 1hour at 80V. The gel was stained with SYBR Safe DNA Gel Stain and visualised on ImageQuant LAS 4000 (GE Healthcare Life Sciences).

**Chromatin immunoprecipitation Sequencing**

K562 cells were cross-linked with 0.4% formaldehyde for 15 minutes at room temperature and with agitation in a glass bottle. To stop the cross-link, glycine (125mM) was added and incubated at room temperature for 10 minutes with agitation. The cell suspension was transferred to a 50mL falcon tube and centrifuged at 515g for 6 minutes at 4°C. The cell pellet was resuspended in 1.5 mL of ice-cold PBS containing 10mM of sodium butyrate, 50µg/mL of phenylmethylsulfonyl fluoride (PMFS) and 1µg/mL of leupeptin to inhibit protease activity and phoshpoSTOP to inhibit phosphatase activity. The cell suspension was centrifuged at 400g for 5 minutes at 4°C and the cell pellets were frozen at -80°C (5x107 cells per pellet).

The K562 cell pellets were thawed on ice and resuspended in nuclear lysis buffer (NLB). This suspension was incubated on ice for 10 minutes and immunoprecipitation dilution buffer (IPDB) was added to obtain the optimal sodium dodecyl sulphate (SDS) concentration for sonication. Sample sonication was performed and optimized in a Bioruptor® Pico (Diagenode) to obtain DNA fragments sheared to approximately 150 to 500 base pairs (bp). After sonication, samples were centrifuged at 16100g for 10 minutes at 4°C. The supernatant was transferred to a new 15mL falcon and IPDB was added to dilute the amount of SDS to 0.2%.

In order to pre-clear the chromatin, protein G-agarose beads were added to the chromatin and incubated on a rotary wheel for 3 hours at 4°C. This suspension was centrifuged at 500g for 5 minutes at 4°C and the supernatant recovered and incubated overnight with the antibodies of interest (≈10µg) on a rotary wheel at 4°C. After this incubation step, pre-blocked protein G-agarose beads were added to the suspension and incubated on a rotary wheel for 3 hours at 4°C. Several washing steps were performed with immunoprecipitation washing buffer 1, 2 (IPWB1, IPWB2) and Tris-EDTA buffer (TE) and a final elution step with immunoprecipitation elution buffer (IPEB) was done to remove the chromatin bound to the antibodies. This DNA suspension was incubated with RNaseA (2µg) and sodium chloride (NaCl – 300mM) for 6 hours at 65°C. To degrade the remaining proteins the DNA suspension was incubated overnight with proteinase K (80µg) at 55°C.

To purify the recovered DNA, a phenol:chloroform:isoamyl alcohol extraction and ethanol precipitation were performed. The purified DNA was quantified using the Qubit dsDNA HS Assay kit (Thermo Scientific) according to manufacturer’s protocol.

ChIP-Seq libraries were prepared with New England Biolabs (NEB) reagents and NEB adapters and primers using manufacturer’s protocol. In brief, this protocol included steps of DNA end-repair, the addition of dA tails, ligation of adapters, and amplification of the library and gel purification of the ChIP-Seq libraries. For DNA ChIP library gel extraction, after cutting the interesting DNA bands from the 2% agarose gel with a clean blade in a blue light transilluminator, the GFX PCR DNA and Gel Band Purification kit was used to purify the DNA according to manufacturer’s protocol. Sequencing preparation was done according to NextSeq Illumina protocol.

**Chromatin immunoprecipitation Sequencing data analysis**

The reads were mapped to the mouse genome using Bowtie ^9^. Peaks were detected against rabbit IgG control using MACS^10^. To check the reliability of the ChIP-seq dataset, we compared the MACS output files containing the peaks for total polymerase and for initiating (S5) polymerase. Elongating (S2) polymerase samples were left out for this analysis due to the peaks being generally wider than those in the *S5* or *total* samples, which makes comparisons not consistent enough. Peaks called by MACS were annotated using R package *ChIPpeakAnno*, and then the IDs of the annotated features were listed. In all samples (control and hypo), at both 0h and 1h, roughly 85-95% of the features that appeared in the initiating polymerase lists were also present in the total polymerase ones (Figure S8). Peaks in different experiments were called as “new peaks” if the peaks had a fold enrichment greater than 15 (for RNA Pol II phosphorylated on Serine2) or 30, a p-value smaller than 10-9 and absence of peaks within 1 kb of that genomic location.

To further explore the data provided by the ChIP-Seq we also used two bioinformatics tools to describe the involvement of transcription factor in the binding of the different forms of RNA Pol II to the specific genomic areas. These tools were PASTAA, a tool from Max Planck Institute at http://trap.molgen.mpg.de and Transcription Factor classification was done at http://tfclass.bioinf.med.uni-goettingen.de/tfclass. In PASTAA we obtained association scores for transcription factors by uploading lists of significantly enriched peaks coming from the ChIP-Seq of total RNA Pol II, PhosphoS5 and PhosphoS2 binding sites. We uploaded Ensembl IDs and used Ensembl gene set_46. For PhosphoS5 (the initiating form of RNA Pol II) we defined promoter regions to be from -200bp to +200bp from the TSS (Transcription Start Site). For PhosphoS2 and total RNA Pol II forms we opened the interrogating region from -10000bp to 0. Full genomic sequence sets were analysed under default calculation of normal affinity.

**Fluorescence Loss in Photobleaching – FLIP**

The assessment of transcription dynamics was also done by a fluorescence loss in photobleaching (FLIP) assay performed in LSM 710 (Carl Zeiss) confocal microscope with the stage heated at 39ºC. For this experiment the modified Chinese hamster ovarian (CHO-K1) cell line^4^ that at 39ºC expresses an RNA Polymerase II-EGFP (RNA Pol II-EGFP) was maintained in DMEM/F12 supplemented with 10% FBS and 100units/mL of penicillin and 100µg/mL of streptomycin. Briefly, this cell line was originated from a mutant CHO-K1 cell line ^5^ with a temperature-sensitive mutation in the largest catalytic subunit of RNA pol II (tsTM4 cell clone). A human RNA Pol II wild-type subunit (hRPB1) was tagged with EGFP and expressed in the tsTM4 cell clone. This construct complemented the defect at the restrictive temperature and enabled the mutant cells to grow normally in contrast to what was observed in the parental cell line ^4^. Under culture conditions at 39ºC the RNA Pol II-EGFP is the main source of transcriptional elongation rather than the endogenous enzyme ^4^.

The CHO RNA Pol II-EGFP cell line was used after 4 days of culture at 39ºC to ensure the stable expression of the RNA Pol II-EGFP form. Cells expressing less RNA Pol II-EGFP in the cytoplasm were chosen to minimize any contribution of nuclear import to fluorescent recovery. A rectangle of half of each nucleus was selected where 100% laser power was applied, in order to bleach all the fluorescent molecules in these rectangles. This operation was repeated approximately every 5 seconds for a period of 900 seconds and the decay of the fluorescence in the unbleached half was analysed.

Fluorescence intensity was analyzed in ImageJ. Fluorescence decay curves were analysed using Sigma Plot 11.0 for Windows and data were fitted to three populations with an exponential decay ($f=a\times\exp\left( -b\times x \right)+c\times\exp\left( d\times x \right)+g*exp(-h\times x)$; R2>0.99): one free form, one bound to DNA but not fully engaged, and another fully engaged in transcription. (see model in Figure S5). FLIP analysis allows to estimate the dissociation constants of the different populations. In order to associate these constants to a functional process, we treat cells with inhibitors of specific steps of the transcription cycle, and then see which kinetic regime is affected. In this way, we can link a decay constant to a specific transcription step ([Hieda et al. 2005](#_ENREF_24), [Sugaya et al. 2000](#_ENREF_54)).

For the fitting to the exponential decays in Figure S5, we allowed the three components to optimize with no restriction (always R^2^>0.99). These fittings allow us to estimate the fraction of the different populations and the rate constants K_2_ and K_4_ (Figure S5). To obtain the other kinetic constants (K_1_ and K_3_) we fitted our data to the steady-state solution of the kinetic model in Figure S5.

**CD34+ UCB isolation**

UCB was collected by the maternity unit from *Hospital Infante D. Pedro Aveiro* in Portugal according to standard collection procedure using a blood bag (SURU). The collected samples were stored at room temperature until processing the samples.

To isolate the specific cell populations within the UCB, flow cytometry cell sorting and magnetic assorted cell sorting techniques were used.

Firstly, the UCB was fractioned to isolate the mononuclear cells, using lymphoprep according to manufacturer’s protocol. The magnetic assorted cell sorting was performed against CD34 and CD133 epitopes according to manufacturer’s protocol. A schematic workflow representation is shown below, where the black arrow in the middle image denotes the layer of mononuclear cells (MNCs) after density gradient fractionation:


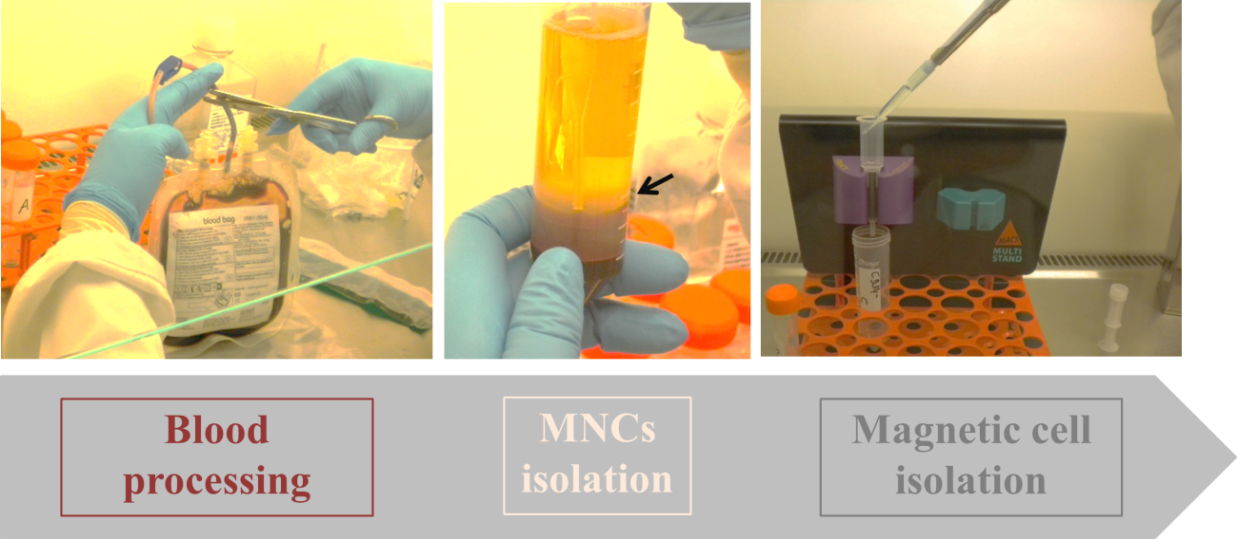


For fluorescence-activated cell sorting, a BD FACS Aria III was used in collaboration with Centro de Histocompatibilidade do Centro and the cells were collected in complete growth medium.

After the cell sorting, the cells were kept overnight in StemSpan supplemented with 100ng/mL of Flt3-L, SCF and 100units/mL of penicillin and 100µg/mL of streptomycin to recover.

**Lentiviral production**

The viral packaging was performed in 293T with the appropriate amounts of transfection agent Lipofectamine 2000 and plasmids of interest. The lipofectamine was mixed with DMEM without serum and incubated at room temperature for 10 minutes. After incubation, the plasmids were added and maintained at room temperature for 30 minutes. Lastly, this mixture was added to a 70%-80% confluent 293T culture flask (T75 flask). The 293T medium was changed by fresh medium after 16 h of incubation (at 37ºC).

Three days after transfection, the 293T viral supernatants were collected into 50mL tubes and spun at 300g for 5 minutes to pellet the cellular debris. After centrifugation, the supernatants were filtered (0,4μm syringe filter) into centrifuge tubes and centrifuged at 19000g for 4 hours. The pellets were then resuspended in 200μL of PBS and stored in aliquots at -80ºC.

**Reprogramming protocol**

The cell reprogramming protocol used was based on the report by Professor Juan Carlos Izpisúa Belmonte ^11^. The viral vector used is a polycistronic lentiviral vector containing c-Myc, Sox2, Oct4, Klf-4 cDNA kindly donated by Professor Axel Schambach ^12^. One day after the last viral infection, the cells were plated on a feeder cell layer of MEFsI. For kinetic studies, colonies growth and reporter fluorescence were monitored by fluorescence microscopy (Axioivert 200M – Carl Zeiss). In these experiments the endpoint was an alkaline phosphatase staining done at day 17 (SIGMAFAST BCIP/NBT). The protocol used is schematized below, where (A) is related with experiments using UCB cells and (B) with experiments using fibroblasts:

**Transdifferentiation protocol**

The transdifferentiation protocol used was based on reports by Professor Thomas Graf group ^2^, whom kindly donated the HAFTL-C10 cell line, the transdifferentiation was induced by the addition of 100 nM of β-estradiol. At the experimental endpoint, the cells were evaluated for CD11b and CD19 epitopes expression by flow cytometry (Gallios) and were also sampled for RNA purification. The protocol used is schematized below:

- **Supplementary References**

1. Amit, M. *et al.* Clonally derived human embryonic stem cell lines maintain pluripotency and proliferative potential for prolonged periods of culture. *Developmental biology* **227,** 271–278 (2000).

2. Bussmann, L. H. *et al.* A Robust and Highly Efficient Immune Cell Reprogramming System. *Cell Stem Cell* **5,** 554–566 (2009).

3. Partikian, A., Olveczky, B., Swaminathan, R., Li, Y. & Verkman, A. S. Rapid diffusion of green fluorescent protein in the mitochondrial matrix. *The Journal of cell biology* **140,** 821–829 (1998).

4. Sugaya, K., Vigneron, M. & Cook, P. R. Mammalian cell lines expressing functional RNA polymerase II tagged with the green fluorescent protein. *Journal of cell science* **113 ( Pt 1,** 2679–2683 (2000).

5. Tsuji, H. *et al.* Isolation of temperature-sensitive CHO-K1 cell mutants exhibiting chromosomal instability and reduced DNA synthesis at nonpermissive temperature. *Somatic Cell and Molecular Genetics* **16,** 461–476 (1990).

6. Iborra, F. J., Escargueil, A. E., Kwek, K. Y., Akoulitchev, A. & Cook, P. R. Molecular cross-talk between the transcription, translation, and nonsense-mediated decay machineries. *Journal of cell science* **117,** 899–906 (2004).

7. Pombo, a *et al.* Regional specialization in human nuclei: visualization of discrete sites of transcription by RNA polymerase III. *The EMBO journal* **18,** 2241–2253 (1999).

8. Iborra, F. J., Jackson, D. A. & Cook, P. R. . R. The path of transcripts from extra-nucleolar synthetic sites to nuclear pores:transcripts in transit are concentrated in discrete structures containing SRproteins. *Journal of cell science* **111,** 2269–2282 (1998).

9. Langmead, B., Trapnell, C., Pop, M. & Salzberg, S. Ultrafast and memory-efficient alignment of short DNA sequences to the human genome. *Genome Biol.* **10,** R25 (2009).

10. Zhang, Y. *et al.* Model-based Analysis of ChIP-Seq (MACS). *Genome Biology* **9,** R137 (2008).

11. Giorgetti, A. *et al.* Generation of induced pluripotent stem cells from human cord blood cells with only two factors: Oct4 and Sox2. *Nature protocols* **5,** 811–820 (2010).

12. Warlich, E. *et al.* Lentiviral vector design and imaging approaches to visualize the early stages of cellular reprogramming. *Molecular therapy : the journal of the American Society of Gene Therapy* **19,** 782–789 (2011).
